# A robust multi-scale approach to quantitative susceptibility mapping

**DOI:** 10.1016/j.neuroimage.2018.07.065

**Published:** 2018-12

**Authors:** Julio Acosta-Cabronero, Carlos Milovic, Hendrik Mattern, Cristian Tejos, Oliver Speck, Martina F. Callaghan

**Affiliations:** a Wellcome Centre for Human Neuroimaging, UCL Institute of Neurology, University College London, London, United Kingdom; b German Center for Neurodegenerative Diseases (DZNE), Magdeburg, Germany; c Department of Electrical Engineering, Pontificia Universidad Catolica de Chile, Santiago, Chile; d Biomedical Imaging Center, Pontificia Universidad Catolica de Chile, Santiago, Chile; e Department of Biomedical Magnetic Resonance, Institute of Experimental Physics, Otto von Guericke University, Magdeburg, Germany; f Center for Behavioural Brain Sciences, Magdeburg, Germany; g Leibniz Institute for Neurobiology, Magdeburg, Germany

**Keywords:** Magnetic susceptibility, Quantitative MRI, Iron mapping, Venography, Variational regularisation, Laplacian pyramid, Quantitative Susceptibility Mapping, (QSM), Multi-Scale Dipole Inversion, (MSDI), nonlinear Morphology-Enabled Dipole Inversion, (nMEDI), High-Pass Susceptibility Mapping, (HPSM), MSDI-based Venography, (VenoMSDI), Macroscopic-Vessel Suppressed Susceptibility Mapping, (MVSSM), HPSM-based Susceptibility-Weighted Imaging, (HPSM-SWI), Maximum Susceptibility Projection, (MSP), spherical mean - value, (SMV), variable SMV, (vSMV), Laplacian boundary value, (LBV), normalised root-mean-square error, (RMSE), high-frequency error norm, (HFEN), structure dissimilarity index, (1-SSIM), mean absolute multi-ROI error, (ROI Error), Calculation of Susceptibility through Multiple Orientation Sampling, (COSMOS), prospective motion correction, (PMC)

## Abstract

Quantitative Susceptibility Mapping (QSM), best known as a surrogate for tissue iron content, is becoming a highly relevant MRI contrast for monitoring cellular and vascular status in aging, addiction, traumatic brain injury and, in general, a wide range of neurological disorders. In this study we present a new Bayesian QSM algorithm, named Multi-Scale Dipole Inversion (MSDI), which builds on the nonlinear Morphology-Enabled Dipole Inversion (nMEDI) framework, incorporating three additional features: (i) improved implementation of Laplace's equation to reduce the influence of background fields through variable harmonic filtering and subsequent deconvolution, (ii) improved error control through dynamic phase-reliability compensation across spatial scales, and (iii) scalewise use of the morphological prior. More generally, this new pre-conditioned QSM formalism aims to reduce the impact of dipole-incompatible fields and measurement errors such as flow effects, poor signal-to-noise ratio or other data inconsistencies that can lead to streaking and shadowing artefacts. In terms of performance, MSDI is the first algorithm to rank in the top-10 for all metrics evaluated in the 2016 QSM Reconstruction Challenge. It also demonstrated lower variance than nMEDI and more stable behaviour in scan-rescan reproducibility experiments for different MRI acquisitions at 3 and 7 Tesla. In the present work, we also explored new forms of susceptibility MRI contrast making explicit use of the differential information across spatial scales. Specifically, we show MSDI-derived examples of: (i) enhanced anatomical detail with susceptibility inversions from short-range dipole fields (hereby referred to as High-Pass Susceptibility Mapping or HPSM), (ii) high specificity to venous-blood susceptibilities for highly regularised HPSM (making a case for MSDI-based Venography or VenoMSDI), (iii) improved tissue specificity (and possibly statistical conditioning) for Macroscopic-Vessel Suppressed Susceptibility Mapping (MVSSM), and (iv) high spatial specificity and definition for HPSM-based Susceptibility-Weighted Imaging (HPSM-SWI) and related intensity projections.

## Highlights

• MSDI mitigates QSM reconstruction artefacts through effective preconditioning.
• MSDI leads to accurate and stable QSM across a broad range of imaging conditions.
• Multi-scale information enables novel contrasts with greater tissue specificity.

## Graphical abstract

**Figure:**
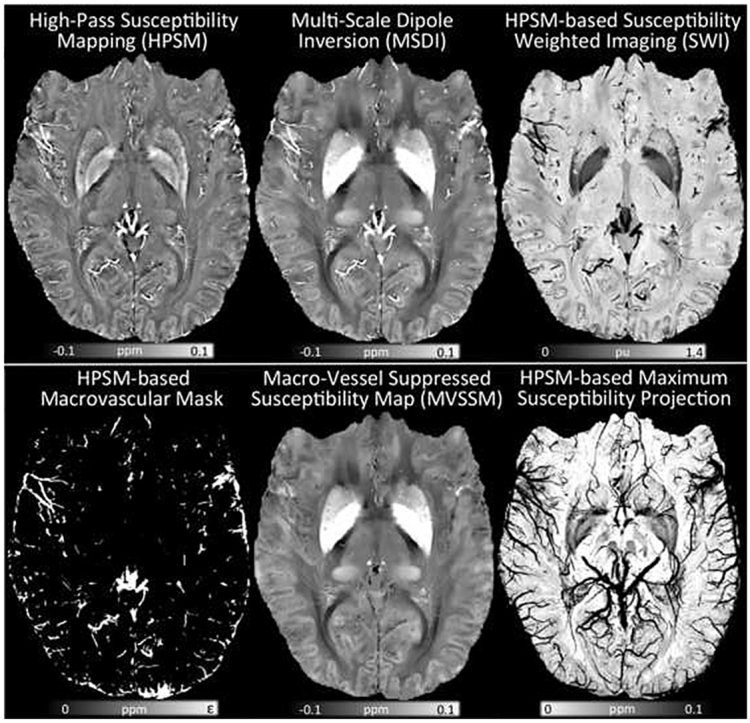

## 1 Introduction

Quantitative susceptibility mapping (QSM) is a descriptor of tissue magnetic susceptibility that has attracted much interest in recent times (Haacke et al., 2015; Wang and Liu, 2015). QSM's potential for clinical neurology and neuroscience applications chiefly lies on its sensitivity to variations in content of brain iron and other chemical species such as lipids (*e.g.* myelin phospholipids) or calcium (Wang et al., 2017). QSM is also known for its sensitivity to differential oxygen-saturation levels in venous blood (Fan et al., 2014), and might also be sensitive to variations across white matter microstructure at the sub-voxel level (Wharton and Bowtell, 2015). QSM has undergone post mortem validation (Langkammer et al., 2012; Sun et al., 2015; Zheng et al., 2013); and has shown exquisite anatomical detail *in vivo*, particularly at ultra-high field (Bian et al., 2016; Costagli et al., 2016; Deistung et al., 2013).

In the field of applications, QSM has successfully been used to detect brain alterations in *e.g.* aging (Acosta-Cabronero et al., 2016; Betts et al., 2016; Darki et al., 2016; Li et al., 2014; Persson et al., 2015), drug addiction (Ersche et al., 2017), Alzheimer's disease (Acosta-Cabronero et al., 2013; Ayton et al., 2017; Moon et al., 2016; van Bergen et al., 2016b), Parkinson's disease (Acosta-Cabronero et al., 2017; Guan et al., 2017; He et al., 2015), Huntington's disease (Dominguez et al., 2016; van Bergen et al., 2016a), neurodegeneration with brain iron accumulation (Lobel et al., 2014), neuromuscular disease (Acosta-Cabronero et al., In Press; Costagli et al., 2016; Schweitzer et al., 2015), demyelinating disease (Bian et al., 2016; Chen et al., 2014; Cronin et al., 2016; Li et al., 2016; Wisnieff et al., 2015; Zhang et al., 2016) and traumatic brain injury (Liu et al., 2016). QSM has also been proposed for vascular monitoring both at single time points (Fan et al., 2015, 2016; Ozbay et al., 2015) or in combination with the MRI technique of arterial spin labelling to infer cerebral metabolic rates of oxygen extraction (Buch et al., 2017; Zhang et al., 2018); and dynamically, using fast echo-planar acquisitions for functional MRI (fMRI) applications (Balla et al., 2014; Bianciardi et al., 2014; Chen and Calhoun, 2015; Ozbay et al., 2016; Sun et al., 2017).

In brief, a typical QSM reconstruction routine requires accurate coil-combination, often followed by phase unwrapping and background-field extraction steps to isolate local-field offsets, which must then be deconvolved (typically using a dipole kernel) to retrieve the underlying tissue susceptibilities. At present, the overwhelming majority of QSM methods formulate this field-to-source inversion problem in Bayesian terms (de Rochefort et al., 2010); that is, trading data/model inconsistencies with an energy functional that promotes some prior knowledge (mathematical and/or anatomical), *e.g.* that the 3D gradient of the susceptibility distributions must be sparse (*e.g.* using $\ell_{1}$-norm based regularisation penalties) or that the solution must share edges with a structural image (*e.g.* by spatially weighting the regulariser). Such priors help condition an otherwise ill-posed inversion operation (Kee et al., 2017; Wang et al., 2013, 2016).

Indeed, variational methods are currently the highest performing algorithms for QSM reconstruction (Langkammer et al., 2018), though importantly, they are not immune to poor pre-conditioning making them notoriously dependent on *e.g.* effective background-field cancellation prior to inversion (Schweser et al., 2017). In order to reduce the dependency on optimal background field removal, further pre-conditioning can be achieved through incorporating harmonic-field estimation principles such as the spherical mean - value (SMV) property or satisfying the relevant Laplace's (or Poisson's) equations within single-step Bayesian formulations (Chatnuntawech et al., 2017; Kee et al., 2017; Langkammer et al., 2015; Liu et al., 2017). Such approaches have successfully demonstrated solid improvements *e.g.* in reducing so-called “shadow” or “shadowing” artefacts (Kee et al., 2017). Notably, at least in their current form, these approaches offer limited error/artefact propagation control beyond the mathematical prior imposed by their variational term; often promoting sparsity in the gradient of the solution to prevent over-fitting, thereby helping to constrain convolution errors introduced by the double-conical distribution of zero dipole-kernel coefficients, i.e. “streaking” artefacts. These methods are therefore somewhat reliant on having relatively consistent input data in order to avoid systematic over-regularisation.

Beyond the action range of variational regularisers, some previously proposed (inversion-only) algorithms have shown relative success in controlling reconstruction artefacts. For example, the nonlinear Morphology-Enabled Dipole Inversion (nMEDI) approach (Liu et al., 2013) capitalises on the following additional strategies: (i) it incorporates a nonlinear consistency term to improve noise management, (ii) it dynamically rejects cost contributions from potentially inconsistent data (to prevent them from dominating the data-fidelity weight), and (iii) it promotes piece-wise constant susceptibility distributions except at locations of strong 3D magnitude gradient, which reduces streaking artefacts and helps better resolve the vasculature and other local features (Kee et al., 2017; Liu et al., 2011, 2012a; Wang et al., 2016).

In practice, however, both approaches are vulnerable to streaking and shadowing artefacts originating from dipole-incompatible fields (Kee et al., 2017). Thus, the goal of this work was to merge and extend both methodological strands with the development of a new Multi-Scale Dipole Inversion (MSDI) algorithm, which, building on the nMEDI framework, implements Laplace's equation by splitting the QSM problem in parts through variable SMV-based filtering and subsequent deconvolution, in turn also conveniently enabling adaptive (spatial-scale dependent) strategies for error control and regularisation, which we hypothesised would result in more robust convergence pathways.

In order to contextualise its performance, the proposed approach (MSDI) was compared with other algorithms in the framework of the 2016 QSM Reconstruction Challenge (Langkammer et al., 2018). We investigated the algorithm's test-retest reproducibility and the reliability of L-curve analysis for free-parameter estimation (Hansen and O'Leary, 1993) using different gradient-echo sequences; single- and multi-echo acquisitions with isotropic and anisotropic image resolutions at different field strengths of 3 and 7 Tesla. Finally, we explored new post-processing strategies that capitalise on MSDI's source-separation ability across spatial scales, with a focus on improving visualisation and tissue specificity relative to conventional QSM and Susceptibility-Weighted Imaging or SWI (Haacke et al., 2004).

## 2 Materials and methods

### 2.1 Multi-scale dipole inversion (MSDI)

Broadly, the proposed algorithm aims to construct a Laplacian pyramid (Burt and Adelson, 1983) via serial bandpass filtering for an improved implementation of Laplace's equation and more adaptive noise modelling. MSDI is thus formulated as a multi-scale variational problem where the initial field map is inverted in parts by modulating the spatial scale of the deconvolution operation. In practice, splitting the full inversion into a sequence of differential sub-problems enables a more selective use of priors and tighter error control moving from short- to long-range dipole field components. The algorithm formulation is specifically set to minimise noise amplification, artefact propagation and mask erosion across scales as described below.

In MSDI the susceptibility distribution, *X*_*l*_, is the sum of susceptibility estimates from *l* pyramid levels (or spatial “scales” – terminology used hereafter), *i.e.* $X_{l}=\sum_{l}X_{l}^{\prime }$, each scale is subject to specific pre-conditioning in the spatial domain such that:

(1) $$\phi_{l}^{'}=\phi_{l}-S_{l}*\phi_{l}=\left(\delta -S_{l}\right)*\phi_{l}\text{,}$$

where *ϕ*_*l*_ and $\phi_{l}^{'}$ represent a phase distribution and its high-pass filtered counterpart, respectively; *S*_*l*_ is the SMV kernel (Schweser et al., 2017) with radius, *r*_*l*_; *δ* represents the Dirac delta function; and * is the 3D convolution operator. Eq. (1) can also be expressed as $\phi_{l}^{'}=F^{H}{\tilde{S}}_{l}F\phi_{l}$, where ${\tilde{S}}_{l}=F\left(\delta -S_{l}\right)$. The latter is the high-pass complement of the SMV kernel in the frequency domain and F is the Fourier transform operator with inverse, F^H^.

Turning to the initialisation of Eq. (1), *ϕ*_*l*_ can be seen as a starting field including all phase contributions unaccounted for in previous scales (or simply the initial phase for the first scale, *l* = 1), *i.e.*:

(2) $$\phi_{l}=\phi -{\text{F}}^{H}D\text{F}X_{l-1}\text{,}$$

where *ϕ* is the initial phase, *X*_*l*-1_ is the susceptibility-sum from the previous (finer) scale (starting from a null matrix for *X*_0_), and *D* is the dipole kernel in the frequency domain (Marques and Bowtell, 2005; Salomir et al., 2003) which includes a *X*-scaling factor to match *ϕ* units. Such an approach enables the use of scale-specific constraints; with the added desirable characteristic that stepwise re-initialisation (Eqs. (1), (2))) prevents noise amplification and mask erosion leading to the following sub-problem:

(3) $$X_{l}^{'}=\underset{X^{'}}{\text{argmin}\;}\lambda {\left\|Q_{l}W_{l}\left(e^{i{\text{F}}^{\text{H}}{\tilde{S}}_{l}D\text{F}X^{'}}-e^{i\phi_{l}^{'}}\right)\right\|}_{2}^{2}+{\left\|M_{l}^{\nabla }\nabla X^{'}\right\|}_{1}\text{.}$$

In Eq. (3), *λ* is the regularisation parameter, ∇ represents the 3D gradient (implemented as a finite-differencing operator), $M_{l}^{\nabla }$ is a scale-dependent edge-mask *i.e.* a dynamic morphological constraint to the regulariser, *W*_*l*_ is a data-driven weighting matrix that adaptively (with updates at each iteration) compensates for *ϕ*′ noise non-uniformities, and *Q*_*l*_ is an additional scale-specific phase-reliability mask that increasingly suppresses consistency cost contributions as a function of the SMV-kernel size.

Eq. (1) and Eq. (3) imply Eq. (2) may be initialised for the first scale (*l* = 1) with an unwrapped phase distribution without additional pre-processing. In practice, however, this study found MSDI benefits from prior background-field elimination using standard methods (details provided in the following subsections).

### 2.2 Dynamic error control

In line with Eqs. (1), (2)), the fidelity term in Eq. (3) defines a scalewise (${\tilde{S}}_{l}$ kernel-size dependent) dipole deconvolution operation that stratifies source inversions by the spatial extent of their associated dipole field, hence enabling more adaptive error control.

*W*_*l*_, in analogy to nMEDI, is initialised as an estimate of the inverse of the *ϕ*′ noise distribution in order to compensate for spatially non-uniform measurement reliability. This noise distribution can be approximated by the inverse of the signal magnitude (Gudbjartsson and Patz, 1995), *A*^−1^ for *ϕ*, and by $A_{l}^{-1}=S_{l}*A^{-1}$ for the second term in Eq. (1). Therefore, the inverse of the composite noise distribution at each scale can be expressed as $A_{l}^{\prime }=\left(a_{l_{ijk}}^{\prime }\right)={\left[{\hat{A}}^{-2}+{\hat{A}}_{l}^{-2}\right]}^{-1/2}$, with *Â* and *Â*_*l*_ normalised by their respective mean over a region/volume of interest (ROI/VOI), Ω, *e.g.* a whole-brain mask.

Subsequently at each iteration, *W*_*l*_ is dynamically downscaled, by the square of the residual value, at locations returning large normalised-consistency residuals, ${\hat{R}}_{l,iter}=\left({\hat{r}}_{l,iter_{ijk}}\right)>f$, greater than a defined threshold, *f*. This approach is equivalent to the previously proposed model-error reduction through iterative tuning (MERIT) method, which prevents unphysical model departures from dominating the forward-consistency cost (Liu et al., 2013). *W*_*l*_ can thus be expressed as:

(4) $$W_{l_{ijk}}=\left\{ \begin{array}{ll} \frac{a_{l_{ijk}}^{'}}{{\hat{r}}_{l,iter_{ijk}}^{2}}, & \forall \left(i,j,k\right):{\hat{r}}_{l,iter_{ijk}}>f\\ a_{l_{ijk}}^{'}, & \text{otherwise} \end{array}\right.\text{.}$$

Furthermore, on the empirical observation that data inconsistencies generate more reconstruction artefacts (such as streaking and shadowing) when using large SMV kernels, we also introduce an empirically determined masking rule (applied to all scales except the initialisation, *l* = 1) to dynamically prevent cost contributions from the top $q\frac{r_{l}}{r_{2}}$ th percentile (P) of the measured-phase second differences, Δ''ϕ (Abdul-Rahman et al., 2007). *Q*_*l*_ can be expressed as:

(5) $$Q_{l_{ijk}}=\left\{ \begin{array}{ll} 0, & \forall \left(i,j,k\right):\Delta ''\phi_{ijk}>{{\text{P}}_{100-q\frac{r_{l}}{r_{2}}}|}_{\Omega }\Delta ''\phi ,\begin{array}{c} l\geq 2 \end{array}\\ 1, & \text{otherwise} \end{array}\right.\text{.}$$

Clearly a potentially intractable number of strategies exist for dynamic error control. In this study, the scales defined by *r*_*l*_ and the masking rule in Eq. (5) were empirically optimised (qualitatively, and quantitatively where possible) within the experimental framework of this study (described below).

### 2.3 MSDI implementation

**Fig. 1:**
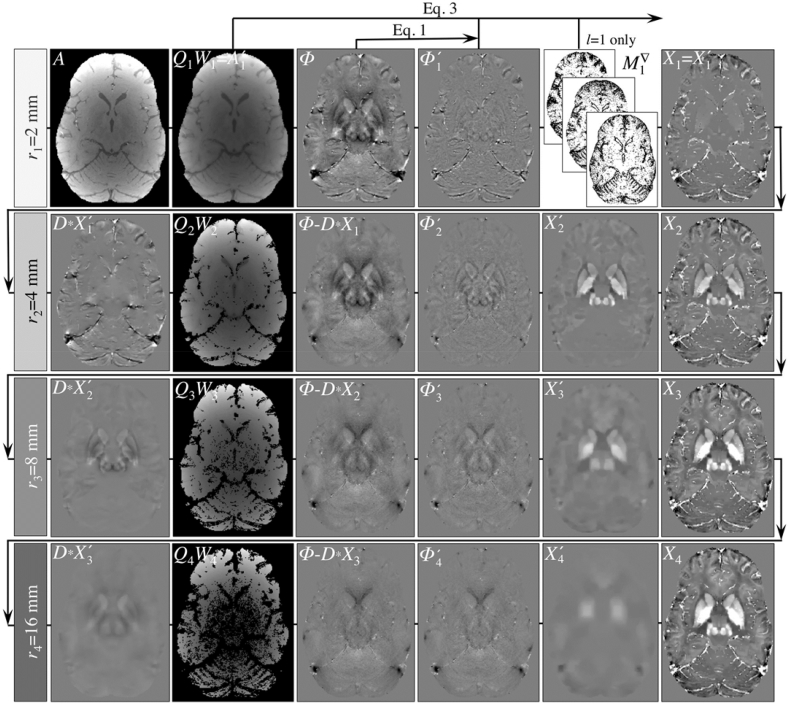
Schematic illustration of the proposed multi-scale dipole inversion (MSDI) method. Each row illustrates the application of Eqs. (1), (2), (3), (4)) across spatial scales. The routine is initialised with SMV filtering with a small kernel radius of 2 mm. The first-scale deconvolution operation uses magnitude priors to ensure accurate depiction of the vasculature and other focal susceptibility gradients if they are co-localised with rapid magnitude variations. Gradually increasing the background-filtering kernel radius in subsequent scales (without using the magnitude prior) gradually recovers sparse susceptibility distributions from increasingly larger-scale fields. In MSDI, to control for the impact of data inconsistencies, a weighting matrix, *W*_*l*_, is applied to compensate for phase-noise non-uniformities in a scale-dependent manner. In addition, the masking rule imposed by *Q*_*l*_ increasingly lowers the threshold for exclusion of noisy phase-neighbourhoods from the data fidelity term.

Fig. 1 schematically illustrates the procedural steps for the proposed MSDI implementation, where each row represents a different spatial scale (or Laplacian-pyramid level).

Through empirical observation (*i.e.* for the experiments described below using a range of *r*_*l*_ settings), we deemed four scales with increasing power-of-two kernel radii (*r*_*l*_ = 2, 4, 8 and 16 mm, or the nearest multiples of voxel resolution) as optimal for the present MSDI implementation. Briefly, contrasting to over-determined solutions for a range of experiments, we observed that large initial SMV radii are greatly inefficient at preventing spurious background contributions to *X*_*l*_, whereas relatively small final radii (*e.g. r*_*l*_ ≤ 6 mm) result in excessively attenuated solutions from a quantitative standpoint. Small SMV radii, however, have merit for some applications, *e.g.* where isolating short-extent dipole fields emanating from the vasculature might be desirable.

Taking this into consideration, the first MSDI scale (row #1 in Fig. 1) is expected to recover susceptibility sources ($X_{1}^{'}$) from highly localised dipole fields ($\phi_{1}^{'}$), *i.e.* those resulting from selective high-pass SMV pre-filtering with a small kernel radius, *r*_1_ = 2 mm (Eq. (1)). Filtered phases are then deconvolved as in Eq. (3); with *W*_1_ initialised as $A_{1}^{\prime }$, *f* = 6 for intra-scale dynamic error control (MERIT default), and applying the anisotropic morphological prior, $M_{l}^{\nabla }$. In keeping with a previous MEDI optimisation, $M_{l}^{\nabla }$ in MSDI was set to mask out the location of the top-30% magnitude gradients (Liu et al., 2012b), though only for the first scale (*i.e. r*_1_ = 2 mm) where vascular features are most prominent, *i.e.* where sharing edges with the magnitude image is a justified prior.

The constrained minimisation problem described in Eq. (3) is somewhat analogous to that in nMEDI (Liu et al., 2013). As such, MSDI's Lagrangian formalism is also linearised to a first-order Taylor expansion and solutions are approximated by the same quasi-Newton fixed-point method provided in the MEDI toolbox (http://weill.cornell.edu/mri/pages/qsm.html), including nested solution updates calculated via conjugate-gradient matrix inversions (Kee et al., 2017). The weak derivative of the $\ell_{1}$-norm term results in a smoothing parameter, *e*, which was set to 10^−6^; default in recent—but not older—versions of the MEDI toolbox. We found this setting ensures conjugate-gradient termination at each quasi-Newton iteration is dictated by a given update step tolerance, not a maximum number of iterations; in this study, *CG*_*tol*_ = 0.1 (MEDI default). In addition, the global stopping tolerance for each scale was set to a normalised residual step size of 0.1 (MEDI default).

Subsequent steps (rows #2–4 in Fig. 1) are expected to gradually recover increasingly lower spatial-frequency features through iteration of Eqs. (1), (2), (3), (4), (5)) with ever-increasing SMV kernel radii. Of note, in this study we chose to keep susceptibility values unreferenced (*i.e.* zero DC-offset throughout).

Turning to Eq. (2), rewriting it for *Φ*_*l-1*_ and by substitution, it can also be expressed as $\phi_{l}=\phi_{l-1}-{\text{F}}^{H}D\text{F}X_{l-1}^{'}$, which more clearly illustrates that the proposed method is in essence a Laplacian pyramid (Burt and Adelson, 1983) that solves Laplace's equation by integrating differential solutions from layers of increasing dipole extent (as defined by *r*_*l*_). In these sub-scales, $M_{l}^{\nabla }$ in Eq. (3) is set to unity across the whole VOI, hence disabling the morphological prior (which acts only on the *l* = 1 sub-scale in the present implementation), whereas consistency contributions from voxels with large local variations (predictive of measurement unreliability) are gradually suppressed as described in Eq. (5) to prevent these inconsistencies from dominating the data fidelity cost function. Through empirical observation and quantitative validation (where possible with experiments described below), the initial masking percentile was empirically set to *q* = 10, *i.e.* the extents of consistency masking across *Q*_2-4_ were 10, 20 and 40%. Inline Supplementary Figs. S1, S2 and S3 illustrate the basis for selecting optimal MSDI model parameters and phase pre-processing in this study.

### 2.4 QSM Reconstruction Challenge assessments

In order to assess its performance, MSDI was compared with nMEDI and other *state-of-the-art* methods in the context of the 2016 QSM Reconstruction Challenge (Langkammer et al., 2018), where 27 algorithms—initialised with single head-orientation data—were set to minimise several error/dissimilarity metrics with respect to the susceptibility tensor component, *χ*_33_. Metrics were: root-mean-square error (RMSE, as a percentage of the absolute ground-truth reference), high-frequency error norm (HFEN) (Ravishankar and Bresler, 2011), structure “dissimilarity” index or 1-SSIM (Wang et al., 2004) and mean absolute error across several grey and white matter regions of interest (ROI Error). In the present study, we also calculated performance metrics relative to a Calculation of Susceptibility through Multiple Orientation Sampling (COSMOS) ground-truth reconstruction (Liu et al., 2009). Subsequently, we assessed the reliability of L-curve analysis (Hansen and O'Leary, 1993) for optimal regularisation parameter estimation through a covariance study of maximum L-curvature versus algorithm performance.

### 2.5 Scan-rescan stability tests

The reproducibility of the proposed algorithm was systematically investigated with data from a single subject (38 y. o. male) who was scanned on a Siemens Trio 3T MRI system using a 32-channel receive array coil (Siemens Healthcare, Erlangen, Germany) on five consecutive days using a 2 × 2-accelerated, spoiled 3D gradient-recalled echo (GRE) sequence with 0.8-mm isotropic voxels (320 × 280 × 224 matrix, head-foot readout direction and 30° field-of-view angulation to minimise the impact of eye-movement artefact in the cortex), 12° flip angle (*α*) and eight bipolar echoes with minimum echo time (*TE*_*min*_), 2.34 ms; inter-echo spacing (Δ*TE*), 2.30 ms; repetition time (*TR*), 25 ms; and receiver bandwidth (*BW*) set to 488 Hz/pixel; giving a total acquisition time (*TA*) of 7:08 min. Hereafter this acquisition will be referred to as “3T Multi-Echo”. Of note, the same positioning routine was adopted for all scans (same head padding, leg-resting cushion, *etc*), but the exact head orientation was not systematically controlled for. This approach was adopted in order to capture the variance that one might expect from serial scanning in normal conditions.

Raw data were reconstructed offline with a sensitivity-encoding algorithm, SENSE (Pruessmann et al., 1999), followed by spatial (best-path) phase unwrapping (Abdul-Rahman et al., 2007), magnitude-weighted least-squares phase fitting with bipolar-readout and transmit-related offset adjustment (see Inline Supplementary Fig. S4), ROI estimation—with the brain extraction tool, BET2 (Smith, 2002), fractional threshold set to 0.1—from the root-mean-square echo-combined magnitude image, and background-phase removal in two steps: LBV (Zhou et al., 2014) with two-layer ROI peeling (otherwise default settings) and vSMV (Li et al., 2011) with *r*_0_ = 40 mm and step-size/final kernel radius of 1 voxel (see Inline Supplementary Fig. S2).

### 2.6 General assessment of acquisition dependencies

We investigated the robustness of the MSDI approach both for 3T and 7T MRI acquisitions. At 3T, in addition to those for “3T Multi-Echo”, summary measures (QSM *mean*/*σ* across repetitions) were also calculated for data acquired with a 2 × 1-accelerated, fully flow-compensated, spoiled 3D GRE pulse sequence (“3T Single-Echo”). These data were acquired five times for the same subject during the same five sessions as the “3T Multi-Echo” data with the following scan parameters: *α* = 13°, single *TE*/*TR* = 20/27 ms, *BW* = 120 Hz/pixel, 256 × 200 × 80 matrix (no field-of-view angulation) with 1 × 1 × 2 mm^3^ voxel resolution giving a *TA* of 4:10 min.

Quantitative susceptibility maps for the “3T Single-Echo” datasets were processed as follows: 3D complex-valued data from each coil element—reconstructed using the Generalised Autocalibrating Partially Parallel Acquisitions (GRAPPA) algorithm (Griswold et al., 2002)—were resliced to 1 mm isotropic resolution via zero-padding. 3D phase maps were then unwrapped with a discrete implementation of the Laplacian method (Schofield and Zhu, 2003), and were subsequently background-filtered using the same two-step harmonic-removal procedure as for “3T Multi-Echo”. This pre-processing step removed coil-specific phase offsets, thus artefact-free, coil-combined filtered phases were inferred from a direct magnitude-square weighted sum.

As for “3T Multi-Echo”, regularisation parameters for inversion were also optimised with L-curve analyses (Hansen and O'Leary, 1993). Regarding spatial normalisation, GRE-magnitude images for each scan repetition were nonlinearly coregistered (using ANTs) to the mean “3T Multi-Echo” magnitude image (in study space), then resulting transformations were applied to each “3T Single-Echo” QSM (with high-order interpolation to 0.8-mm isotropic resolution). These maps were finally brain-masked to enable direct comparisons between the two scan types. In addition to whole-brain maps, mean QSM values were also extracted bilaterally from selected ROIs – globus pallidus, putamen, caudate nucleus, thalamus, hippocampus, amygdala, and mid-sagittal corpus callosum (CC). All ROIs were segmented using previously validated coregistration-based methods (Acosta-Cabronero et al., 2016; Betts et al., 2016).

MRI scanning at high field was performed on Magdeburg's 7T whole-body system (Siemens Healthcare, Erlangen, Germany) in conjunction with a quadrature-transmit/32-channel receive-array coil (Nova Medical Inc., Wilmington, Massachusetts, USA). In this experiment, we evaluated the consistency of three high-resolution acquisitions – all fully flow-compensated, spoiled 3D GRE scans with voxel resolutions 0.33 × 0.33 × 1.25 mm^3^, 0.35 × 0.35 × 1.25 mm^3^ and 0.5 × 0.5 × 0.5 mm^3^ respectively. Imaging parameters for the 0.35 × 0.35 × 1.25 mm^3^ acquisition were *α* = 10°, *TE*/*TR* = 10/18 ms, *BW* = 100 Hz/pixel, 640 × 560 × 120 matrix (no angulation) with 2 × 1 acceleration and further 7/8 partial Fourier sub-sampling along both phase-encoding directions giving a *TA* of 8:46 min. Of note, this sequence has been adopted by EUFIND - a European Union funded consortium concerned with harmonising 7T MRI methods for neurodegenerative disease research and clinical trials (http://www.neurodegenerationresearch.eu/wp-content/uploads/2016/06/JPND_Project-Fact-Sheet_EUFIND-2.pdf), hence in the remaining sections this acquisition will be referred to as “ 7T EUFIND Aniso”. In contrast, both the 0.33 × 0.33 × 1.25 mm^3^ and 0.5-mm isotropic scans used a modified 3D GRE sequence with prospective motion correction (PMC) functionality. Briefly, an in-bore camera (Metria Innovation, Milwaukee, Wisconsin, USA) was used to track a Moiré phase marker attached to the subject's upper jaw (with a custom-made mouthpiece), which enabled short-latency updates to the imaging volume's position and orientation for coherence with prospectively tracked head motion. More details on the PMC system (Maclaren et al., 2012; Stucht et al., 2015) and high-resolution examples can be found elsewhere (Lusebrink et al., 2017a; b; Mattern et al., 2018). These two scans will subsequently be referred to as “7T PMC Aniso” and “7T PMC 0.5Iso”, respectively. Additional scan details for “7T PMC Aniso” were *α* = 10°, *TE*/*TR* = 9.1/20 ms, *BW* = 120 Hz/pixel, 608 × 504 × 88 matrix (full k-space acquisition, no angulation), *TA* = 17:30 min; and for “7T PMC 0.5Iso” were *α* = 10°, *TE*/*TR* = 9.2/20 ms, *BW* = 100 Hz/pixel, 416 × 336 × 288 matrix (no angulation) and 2 × 1 parallel acceleration giving a *TA* of 17:34 min.

Each high-resolution 7T scan was acquired once for the same 35 y. o. male volunteer during different imaging sessions. Uncombined data were exported offline and reconstructed using the same processing routine used for the “3T Single-Echo” data. In order to ease comparability, susceptibility maps were spatially standardised using an ANTs-based procedure (Acosta-Cabronero et al., 2016) with interpolation to the smallest overall voxel volume, *i.e.* 0.5-mm isotropic resolution giving 0.125 mm^3^ voxels. Finally, a global mask was applied to restrict error calculations to the ROI-mask intersection common to all three scan-types. The prediction for this experiment was that robust 7T MSDI should lead to relatively small deviations from the global mean across scan types.

### 2.7 Pilot investigation of MSDI-related contrasts

Focusing on a high-resolution dataset (“7T PMC Aniso”), which due to its highly anisotropic voxel resolution previous modelling work predicts should be highly consistent with vascular dipole fields (Deistung et al., 2008), we explored whether the MSDI approach could produce susceptibility-based MRI contrasts with greater tissue specificity. In other words, we explored whether the multi-scale nature of the present QSM approach could be utilised to isolate tissue types with specific dipole-extent characteristics:

#### 2.7.1 High-pass susceptibility mapping (HPSM) and MSDI-based venography (VenoMSDI)

Close inspection of MSDI-optimisation stages revealed two clear behaviours. First, (i) combined dipole/SMV deconvolution (Eq. (3)) using small spherical-kernel radii (*r*_max_ up to about 4 mm) results in high-pass filtered QSM solutions with excellent anatomical detail. The concept of short-range, high-pass QSM will be referred to hereafter as HPSM. Second, (ii) strong regularisation is very effective at isolating susceptibilities from deoxygenated blood products due to the well-known sparsity promoting properties of Eq. (3). We subsequently hypothesised that (i) with *r*_max_ = 2 mm, in combination with (ii), should essentially capture the continuous, highly intense and spatially restricted field-offset characteristics driven by venous blood vessels. With *r*_max_ = 2 mm, *λ* = 10^1.6^ and binarising positive-negative susceptibilities, we obtained highly confluent masks of the vasculature that enables extracting full-scale MSDI values for venography (VenoMSDI).

#### 2.7.2 Macro-vessel suppressed susceptibility mapping (MVSSM)

With a map of the vasculature, we also derived a complementary MSDI-based contrast for the study of nonheme iron and extravascular blood deposits with greater specificity, *i.e.* MVSSM. The simplest form of MVSSM is the element-wise product of a fully inverted MSDI map and the complement of the binary mask derived for VenoMSDI. This operation, in contrast to that for VenoMSDI, resets all macro-vessel related QSM hypointensities to zero, which we hypothesise is desirable both for greater tissue specificity in regional extractions, and for more robust behaviour in whole-brain statistical analyses requiring spatial smoothing.

#### 2.7.3 HPSM-based Susceptibility-Weighted Imaging (HPSM-SWI)

Furthermore, we explored the possibility of combining HPSM with magnitude information as an alternative to conventional SWI. To this end, we normalised HPSM (*r*_max_ = 2 mm) as described in Eq. (6) below, raised it to the power of four inherited from SWI (Haacke et al., 2009), and used this to weight the RF-bias corrected (Tustison et al., 2010) GRE-magnitude image. HPSM-based weights, Γ_*ijk*_, were inferred as follows:

(6) $$\Gamma_{ijk}=\left\{ \begin{array}{ll} 0, & \forall \left(i,j,k\right):\chi_{1_{ijk}}>c\\ \frac{c-\chi_{1_{ijk}}}{c} & \forall \left(i,j,k\right):0\leq \chi_{1_{ijk}}\leq c\\ 1, & \text{otherwise} \end{array}\right.\text{,}$$

where *c*—set in this study to 0.2 ppm—is a critical value below which (down to zero-susceptibility) high-pass filtered susceptibilities are re-scaled between 0 and 1, and above which Γ_*ijk*_ are set to zero for maximal susceptibility weighting. A highly regularised (*λ* = 10^1.6^) HPSM solution was chosen for creation of this weighting mask. A high degree of smoothness was preferred to prevent noise amplification on magnitude multiplication, whilst the magnitude-based prior (used in combination with the smallest kernel radius) ensured at least some degree of data fidelity for a distribution of spatially concordant rapid field/magnitude variations (*i.e.* those excluded by $M_{l}^{\nabla }$ from the regularising term in Eq. (3)), which almost exclusively captures the venous vasculature. In this study, a qualitative comparison was performed between HPSM-SWI and conventional SWI from the same dataset (“7T PMC Aniso”). Finally, in order to enhance vessel continuity and improve overall visualisation of blood products we also qualitatively explored the potential use of maximum intensity projections from optimally regularised HPSM (MIP-HPSM) as a viable alternative to intensity projections derived from magnitude-weighted contrasts (as in standard SWI).

## 3 Results

### 3.1 QSM Reconstruction Challenge

**Fig. 2:**
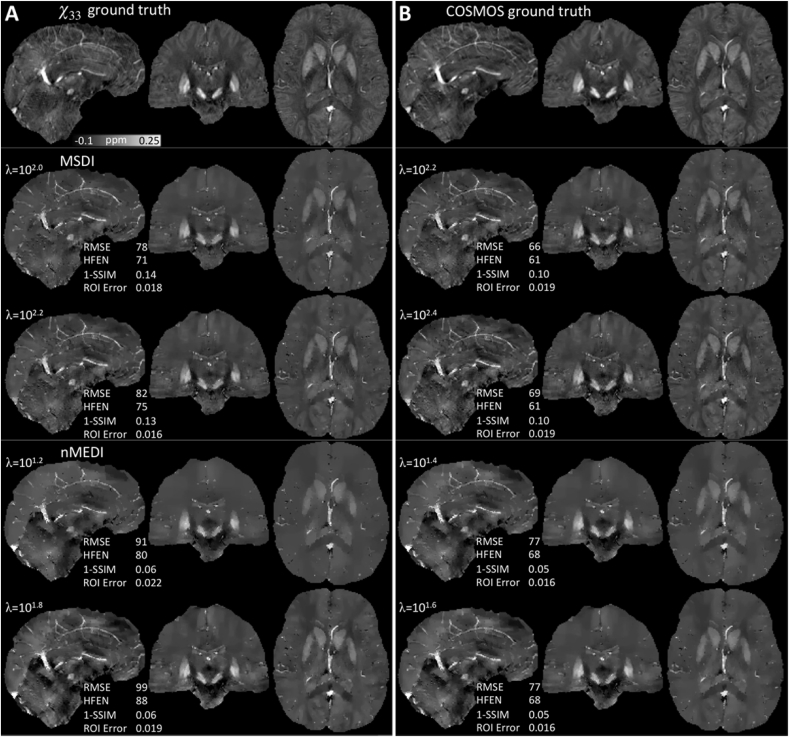
Challenge results. Optimal MSDI (middle row) and nMEDI solutions (bottom row) minimising different performance metrics with respect to (A) *χ*_33_ and (B) COSMOS ground truths (both shown on the top row). The QSM range was clipped to [-0.1, 0.25] ppm for consistency with the Challenge report (Langkammer et al., 2018).

Using data provided for the 2016 Challenge and *χ*_33_ as the ground truth, we found some degree of variability in “optimal *λ”* values for the different performance metrics (Table 1, Fig. 2A). Scores using COSMOS (except ROI Error) indicated better matching than when using the *χ*_33_ reference (Table 1, Fig. 2B). Overall, MSDI returned lower scores than nMEDI for RMSE and HFEN metrics. Using the *χ*_33_ reference specifically, MSDI also outperformed nMEDI for ROI Error, whereas nMEDI reconstructions were preferred according to the 1-SSIM metric. In contrast, using COSMOS, although the 1-SSIM behaviour remained unchanged, nMEDI returned ROI Error levels equivalent to those observed for MSDI with *χ*_33_ while MSDI values were increased. Qualitatively we noted that MSDI yielded more structured reconstructions than nMEDI, which overall returned more severely over-regularised maps.

**Table 1:** Challenge results. Optimal MSDI and nMEDI regularisation parameter values minimising different performance metrics with respect to *χ*_33_ and COSMOS ground truths. Lower metric scores indicate better performance.

	*χ*_33_ ground truth		COSMOS ground truth	
	MSDI	nMEDI	MSDI	nMEDI
RMSE (%)	78 (*λ* = 10^2.0^)	90 (*λ* = 10^1.0^)	66 (*λ* = 10^2.2^)	77 (*λ* = 10^1.4^)
HFEN (%)	71 (*λ* = 10^2.0^)	80 (*λ* = 10^1.2^)	61 (*λ* = 10^2.4^)	67 (*λ* = 10^1.6^)
1-SSIM	0.13 (*λ* = 10^2.2^)	0.01 (*λ* = 1)	0.10 (*λ* = 10^2.2^)	0.02 (*λ* = 1)
ROI Error	0.016 (*λ* = 10^2.2^)	0.019 (*λ* = 10^1.8^)	0.019 (*λ* = 10^2.4^)	0.016 (*λ* = 10^1.8^)

Abbreviations: Root-mean-square error (RMSE, as a percentage of the absolute ground-truth reference), high-frequency error norm (HFEN), structure “dissimilarity” index (1-SSIM), mean absolute error across several grey and white matter regions of interest (ROI Error).

**Fig. 3:**
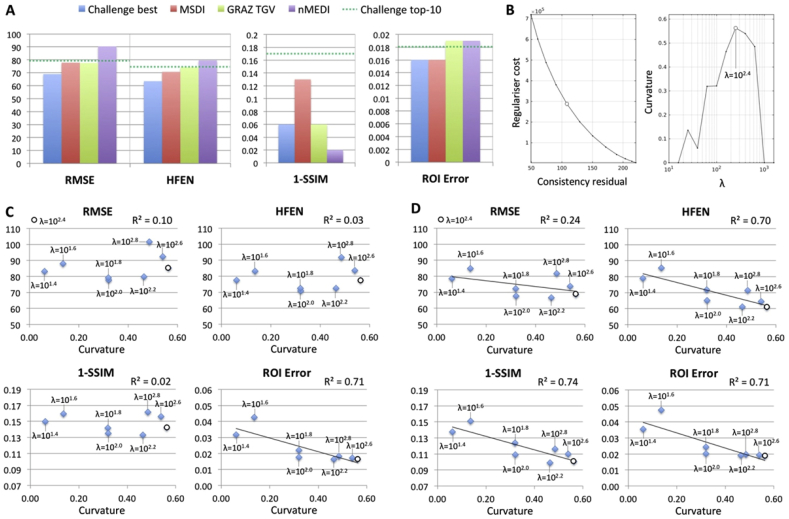
Challenge results. (A) MSDI's relative performance using the *χ*_33_ ground truth. Lower values indicate better performance. “Challenge best” denotes the best method for each metric (*i.e.* metric-specific best). Top-10 algorithms in the Challenge scored 79.1% (RMSE), 74.2% (HFEN), 0.17 (1-SSIM) and 0.018 (ROI Error) or below. GRAZ TGV denotes performance for a Total Generalised Variation (TGV) single-step method (Langkammer et al., 2015) that entered the QSM Workshop Challenge. (B) MSDI's L-curve analysis results (“optimal *λ*”, *i.e.* point of maximum curvature, denoted by an open circle). Algorithm performance as a function of maximum L-curvature using (C) *χ*_33_ and (D) COSMOS ground-truth references.

In contrast to all other methods previously evaluated within the QSM Challenge framework, MSDI ranked in the top-10 for all performance metrics (Fig. 3A). Unexpectedly, however, in this study we found the SSIM-based measure to favourably rank nMEDI reconstructions with little texture (at odds with subjective visual appeal). For example, the best score was achieved with an excessively regularised *λ* = 1 setting. MSDI optimisations, conversely, returned largely consistent behaviours throughout, while still ranking within the Challenge top-10 level for this measure.

Subsequent L-curve analysis (Fig. 3B) returned an optimally cost-balanced *λ* for MSDI that was in close agreement with the metric-optimised *λ*s (using the *χ*_33_ ground truth). However, except for “ROI Error”, we found no evidence to suggest L-curvature co-varied with metric scores (Fig. 3C). In contrast, using the COSMOS ground truth we found L-curvature to be a more robust predictor of MSDI's relative performance (Fig. 3D). Interestingly, we also observed that performance metrics overall favoured over-regularised solutions (relative to L-curve estimates), *i.e.* slightly lower-than-optimal *λ*s returned systematically lower metric scores than higher-than-optimal *λ*s (Fig. 3C–D). Note that *λ* is a multiplicative factor operating on the consistency term in Eq. (3), thus smaller *λ*s lead to greater spatial smoothness, *i.e.* greater sparsity on the 3D gradient of the QSM solution.

### 3.2 Scan-rescan stability

**Fig. 4:**
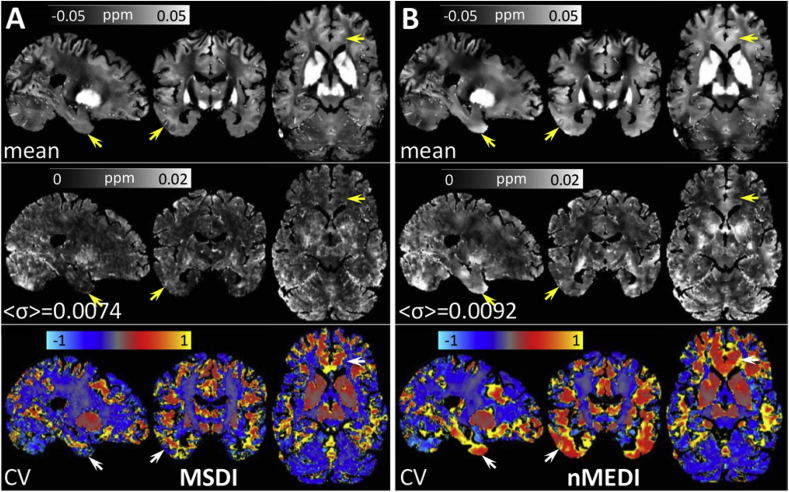
(A) MSDI and (B) nMEDI summary statistics for a reproducibility experiment in which the same “3T Multi-Echo” sequence was used to scan the same 38 y. o. healthy male subject on five consecutive days. (Top row) Representative sagittal, coronal and axial slices for mean QSM over five time-points. (Middle row) Standard deviation (*σ*) over the same five time points, with <*σ*> inset representing the average σ across the whole brain. (Bottom row) Coefficient of QSM variation across repetitions (*CV* = *σ/mea*n). Arrows indicate regions of greater unexplained variation for nMEDI than for MSDI. Though for simplicity arrows are shown unilaterally, regions of high variance are typically bilateral. The opposite behaviour (greater variation for MSDI) was not observed.

Overall, the MSDI and nMEDI methods (both L-curve optimised) yielded visually appealing susceptibility maps from “3T Multi-Echo” data (see Inline Supplementary Fig. S5). On close inspection, however, we noted that MSDI reconstructions yielded greater anatomical detail, with more uniform and more reproducible appearance than nMEDI. The latter was confirmed by direct scan-rescan QSM variability measurements (*σ* across the five repetitions for each method), which showed a 24% increase (on average across the whole brain) for nMEDI relative to MSDI (Fig. 4). This increase in data dispersion was driven by nMEDI instabilities in the corpus striatum, prefrontal white matter, cerebellar grey matter, and across cerebral cortex in orbitofrontal, superior prefrontal, temporal and occipital regions. In contrast, QSM measurements in parietal cortex, primary somatosensory/motor fields and central white matter were the most consistent across methods.

**Fig. 5:**
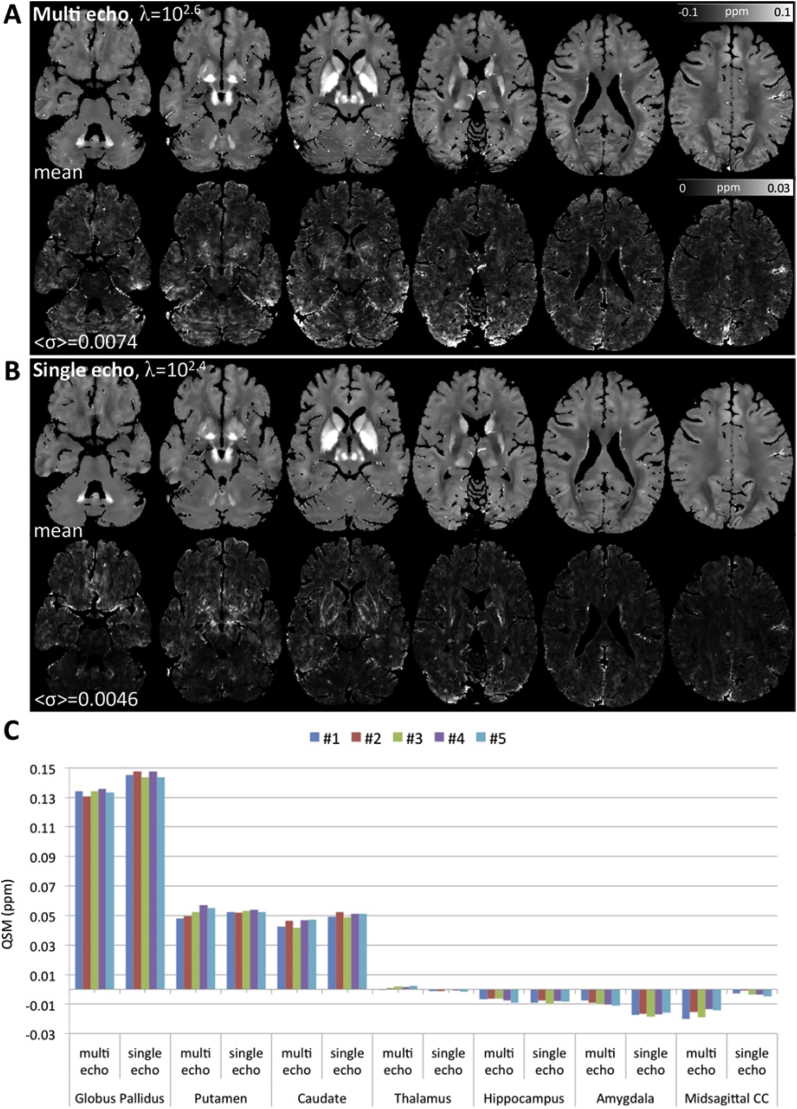
(A) “3T Multi-Echo” and (B) “3T Single-Echo” MSDI reproducibility test results for data from the same subject scanned on five consecutive days with both acquisitions. In each panel, top and bottom rows represent axial cuts for the QSM mean and standard deviation (*σ*) across time-points respectively. (C) Regional study for the same data; each colour bar represents the median QSM for a given scan-type and time-point. Abbreviation: corpus callosum (CC).

### 3.3 Acquisition dependencies

L-curve analyses for the five “3T Multi-Echo” and the five “3T Single-Echo” scan repeats were highly consistent (see Inline Supplementary Figs. S6A–B). The most frequent, optimally cost-balanced *λ* for “3T Multi-Echo” was 10^2.6^ (four times, 10^2.2^ only once), whereas for “3T Single-Echo” the predominant outcome was *λ* = 10^2.4^ (four times, 10^2.6^ only once). In study space, RMSE, HFEN and 1-SSIM scores were calculated between the mean (across repeats) “3T Multi-Echo” QSM, using the L-curve optimised *λ* of 10^2.6^, and the mean (across repeats) “3T Single-Echo” QSM calculated with variable *λ*. The RMSE results are shown in Inline Supplementary Fig. S6C (analogous behaviours were obtained for HFEN and 1-SSIM scores, not shown). The *λ* value for which error scores were minimal (*i.e.* the “3T Single-Echo” reconstruction most consistent with the independently optimised “3T Multi-Echo” QSM reference), matched the global (median) estimate from the L-curve analysis, *i.e. λ* = 10^2.4^, of the “3T Single-Echo” data.

**Fig. 6:**
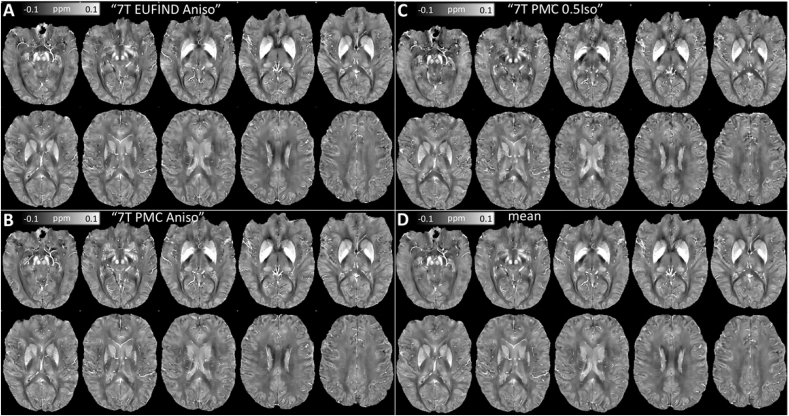
Representative axial slices for MSDI reconstructions of: (A) “ 7T EUFIND Aniso”, (B) “7T PMC Aniso”, and (C) “7T PMC 0.5Iso” data in a common space. (D) Mean macroscopic susceptibility across scan types.

L-curve analyses for high-resolution 7T data were also consistent throughout, converging on an optimal *λ* of 10^2.7^ (see Inline Supplementary Fig. S7). Subsequent qualitative comparisons across 7T scan sub-types (Fig. 6) revealed MSDI is generally stable with high-field data. Although direct comparisons with the overall mean map (Fig. 6D) highlighted a number of differences (see Inline Supplementary Fig. S8): the dispersion was increased for the isotropic data, which, generally, showed slightly greater inconsistencies, particularly in deep brain regions. Nonetheless, departures from the mean were overall small relative to absolute QSM values. Global RMSE scores were similar (RMSE≤62% with respect to the global mean across scan types) for 7T data (using different native image resolutions) to those reported for the fully consistent—but signal-starved—Challenge dataset (RMSE≥66% with respect to a COSMOS ground truth).

### 3.4 MSDI-related contrasts

#### 3.4.1 High-pass susceptibility mapping (HPSM)

**Fig. 7:**
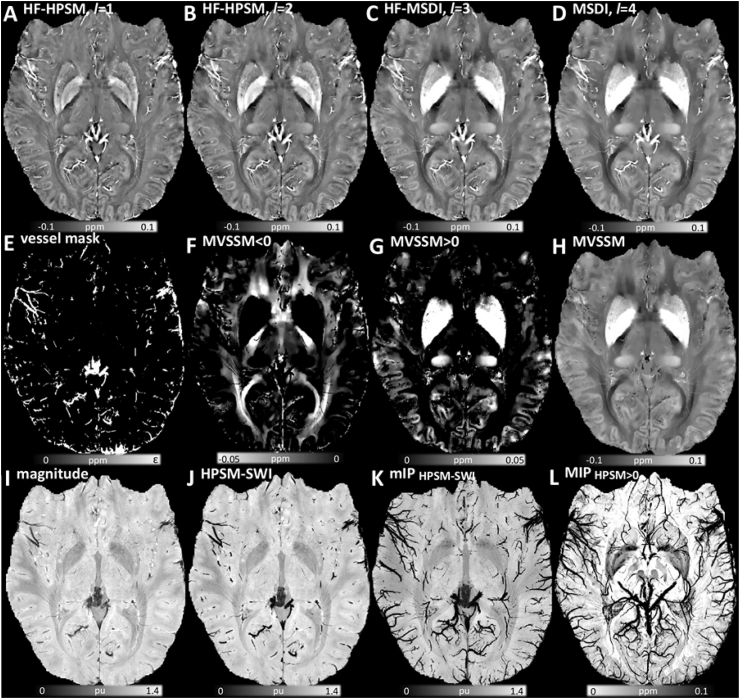
Sample illustration of MSDI-related contrasts from “7T PMC Aniso” data. High fidelity (HF) HPSM: (A) *r*_max_ = 2 mm, *λ* = 10^3.3^. (B) HF-HPSM, *r*_max_ = 4 mm, *λ* = 10^3.1^. (C) HF-MSDI, *r*_max_ = 8 mm, *λ* = 10^2.9^. (D) Full-scale MSDI, *r*_max_ = 16 mm, *λ* = 10^2.7^. (E) Positive-only mask from highly regularised HPSM, *r*_max_ = 2 mm, *λ* = 10^1.6^ (*i.e.* binary mask of the macro-vasculature). (F) Negative-only distribution for MVSSM. (G) Positive-only MVSSM distribution. (H) Full-range MVSSM. (I) RF-bias corrected magnitude image normalised to the whole-brain mean. (J) Highly regularised HPSM based SWI, *r*_max_ = 2 mm, *λ* = 10^1.6^. (K) Minimum-intensity projection map (mIP over 7.5 mm) for HPSM-SWI. (L) Maximum-intensity projection (MIP over 15 mm) for optimally regularised HPSM, *r*_max_ = 2 mm, *λ* = 10^2.7^ (note reversed colour scale for consistency with conventional mIP_SWI_ contrast). Abbreviations: MSDI (Multi-Scale Dipole Inversion), *r*_*l*_ (kernel radius defining *S*_*l*_ and its complement in Eqs. (1), (2), (3))), *λ* (regularisation parameter in Eq. (3)), HPSM (High-Pass Susceptibility Mapping), MVSSM (Macro-Vessel Suppressed Susceptibility Mapping), HPSM-SWI (HPSM-based Susceptibility Weighted Imaging).

**Fig. 8:**
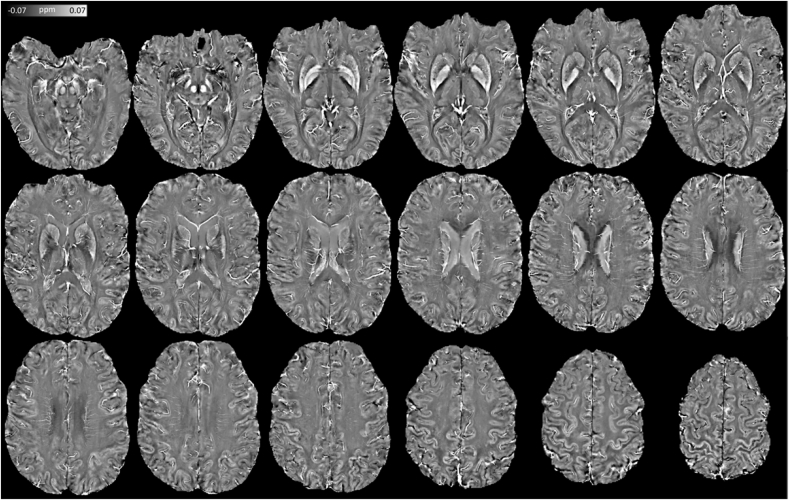
Extended view of high fidelity (*λ* = 10^3.3^), high-pass (*r*_max_ = 2 mm) susceptibility mapping (HF-HPSM) using “7T PMC Aniso” data.

Interestingly, although the same *λ* (*λ* = 10^2.7^) returned maximum L-curvature across all “7T PMC Aniso” MSDI sub-scales, we also obtained highly appealing shorter-range inversions when imposing greater data fidelity to the solution, *e.g.* linearly increasing log_10_(*λ*) with decreasing *r*_max_ = 8, 4 and 2 mm, respectively (Fig. 7A–C). Fig. 8 shows an extended view of the data in Fig. 7A (high fidelity inversion from high-pass SMV-filtered field inductions) with a tighter clipping range to enhance structural detail.

#### 3.4.2 MSDI-based venography (VenoMSDI)

Consistent with our prior hypothesis, we also found that the spatial distribution of highly regularised (*λ* = 10^1.6^, *r*_max_ = 2 mm) positive HPSM values was highly co-localised with the macroscopic vasculature (Fig. 7E).

#### 3.4.3 Macro-vessel suppressed susceptibility mapping (MVSSM)

**Fig. 9:**
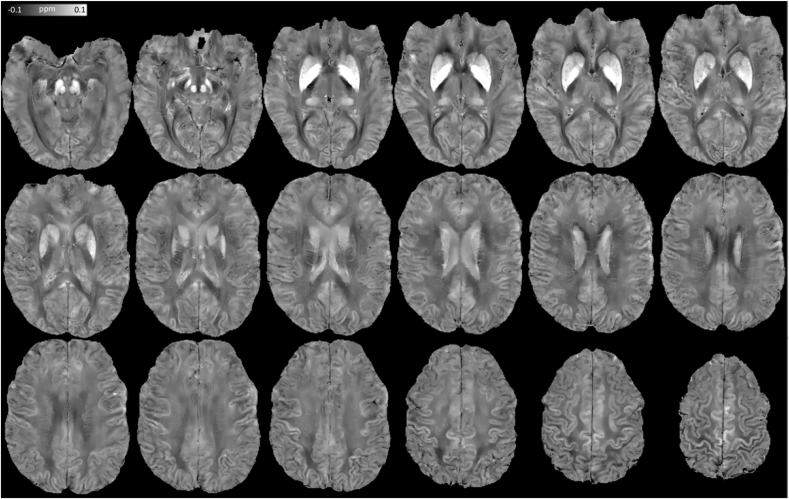
Extended view of optimally regularised (*λ* = 10^2.7^), full-scale (*r*_1-4_ = 2, 4, 8, 16 mm), macro-vessel suppressed susceptibility mapping (MVSSM) from “7T PMC Aniso” data.

The above meant we could readily suppress large blood vessels from QSM using a direct masking procedure (see Fig. 7H versus Figs. 7D, and Fig. 9 for MVSSM's extended view). Formally characterising the efficiency of vessel masking in MVSSM, we contrasted maximum-MVSSM to maximum-MSDI projections over 7.5 mm, which confirmed the high specificity of the proposed method to exclude vascular contributions (see Supplementary Fig. S9).

#### 3.4.4 HPSM-based Susceptibility-Weighted Imaging (HPSM-SWI)

**Fig. 10:**
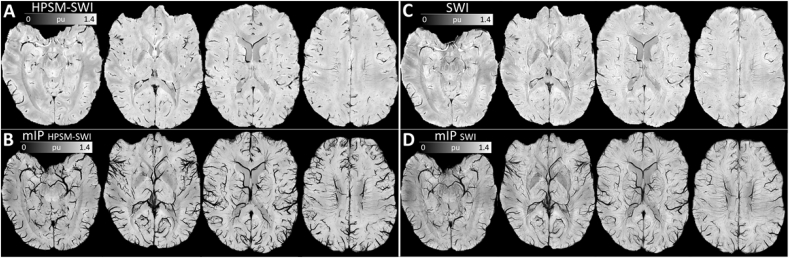
Representative axial slices of (A) highly regularised HPSM-based SWI (HPSM-SWI, *r*_max_ = 2 mm, *λ* = 10^1.6^), and (B) minimum-intensity projections (mIP) over 7.5 mm. Same view for (C) conventional SWI (using a 300 × 300 2D Hanning kernel – the smallest window size for which wrapping errors were not introduced in the weighting mask), and (D) mIP-SWI over 7.5 mm. All maps were inferred from the same dataset (“7T PMC Aniso”), and were normalised by the global mean magnitude across the whole brain prior to post-hoc susceptibility weighting.

Additionally, we explored the use of HPSM as an alternative to high-pass filtered phase maps conventionally used in SWI. This resulted in a qualitatively sharp susceptibility contrast (Fig. 7J–K), which compared positively with conventional SWI on a visual assessment of minimum-intensity projections using the same multiplicative weighting factor of 4 (Fig. 10).

**Fig. 11:**
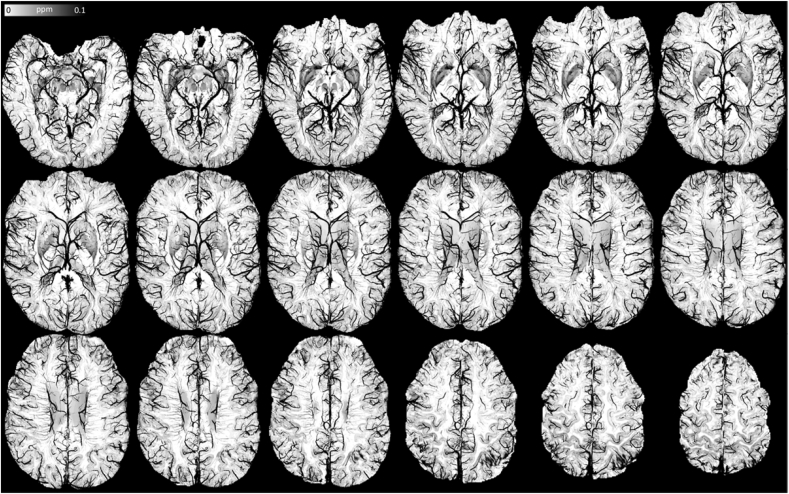
Extended view of the maximum-intensity projection over 15 mm from optimally regularised HPSM (*r*_max_ = 2 mm, *λ* = 10^2.7^) using “7T PMC Aniso” data. Note reversed colour scale, *i.e.* high susceptibility values are hypointense, for consistency with mIP-SWI.

Furthermore, for enhanced visualisation, we confirmed the potential of maximum-intensity projections from optimally regularised HPSM as an alternative to conventional magnitude-based intensity projections (Fig. 7, Fig. 11).

## 4 Discussion

Recent developments proposed the integration of background-removal principles within single-step formulations both for algorithmic efficiency and to improve reconstruction stability (Chatnuntawech et al., 2017; Kee et al., 2017; Langkammer et al., 2015; Liu et al., 2017). However, in their current form vulnerability to artefactual streaking propagation, due to insufficient phase-noise/error considerations, remains. We hereby formulated a Multi-Scale Dipole Inversion (MSDI) approach based on a previously proposed nonlinear dipole inversion solver (Liu et al., 2013), which has been extended through a pyramidal implementation of Laplace's equation to better model background-field remnants, and enable tighter error control via scalewise consistency rejection and more adaptive regularisation through selective edge masking, in turn driving robust convergence pathways that led to accurate and highly reproducible reconstructions for a broad range of different data types.

In the first instance we assessed MSDI's performance in the context of the 2016 QSM Reconstruction Challenge (Langkammer et al., 2018), where unlike any other algorithm proposed to date, it ranked in the top-10 for all performance metrics evaluated. The Challenge consisted of reconstructing a single-orientation dataset from a highly accelerated (*i.e.* relatively signal-starved) acquisition. Interestingly, slightly different regularisation parameters minimised different error metrics using the susceptibility tensor component, *χ*_33_, as the ground truth. The optimal regularisation parameter was more convergent (and consistent with L-curve analysis) when using a COSMOS ground-truth reference instead. Such results provide evidence not only in support of COSMOS as a more suitable target reconstruction for algorithm performance evaluations, but also of L-curvature maximisation as a valid regularisation parameter selection method for MSDI.

Another interesting observation from this experiment was that compared with nMEDI, *i.e.* the backbone of the present multi-scale implementation, MSDI yielded susceptibility maps with greater anatomical detail and improved ground-truth consistency. The latter was true for all performance metrics except for the so-called structure dissimilarity index or 1-SSIM, which was greatly reduced for physically implausible over-regularised nMEDI reconstructions (*λ* = 1). Such unexpected (and undesirable) behaviour is worth highlighting because, although for the MSDI evaluation 1-SSIM was largely concordant with all other metrics, nMEDI's abnormal behaviour speaks against the reliability of 1-SSIM scores and indicates that this metric should be interpreted with caution in this context.

Furthermore, maximum L-curvature versus Challenge-performance plots (see Fig. 3) revealed further interesting behaviour arguing against the commonly held view that slight under-regularisation should be preferred to slightly over-regularised QSM. According to the present Challenge results, error/artefact propagation via under-regularised over-fitting might signify a greater penalty to global QSM accuracy than slight over-compensation. In other words, it might be that QSM regularisation is only sufficient once streaking artefacts and other large-scale features, *e.g.* spurious background remnants, have been tightly constrained, which for algorithms with limited error control or cases with poor (*e.g.* signal-starved or motion-corrupted) input data might result in optimal solutions that are unphysically smooth in appearance.

Despite the immense promise that QSM has shown in the broad array of research and clinical applications listed in the introduction, proportionally less has been reported to date regarding *e.g.* its scan-rescan reproducibility (Deh et al., 2015; Feng et al., 2017; Hinoda et al., 2015; Lauzon et al., 2017; Lin et al., 2015; Santin et al., 2017). Systematic evaluations of this type are particularly pressing given the current need to define a normative baseline over and above which clinicians can seek to identify disease-specific signatures in the spatial distribution of QSM alterations, and the emerging evidence that QSM may be differentially sensitive to pathology, *e.g.* across many neurodegenerative disease states. In addition to the specifics of the data acquisition—*e.g.* number of echoes acquired, field strength used and image resolution—the choice of QSM reconstruction routine (each of which have differential sensitivity to factors such as data inconsistencies or imperfect background field removal) can also influence the scan-rescan reproducibility.

In the present study we demonstrated improved measurement stability with the MSDI approach with respect to nMEDI. It is well known that error/artefact propagation emanating from large and abrupt field offsets, *e.g.* in anterior temporal and orbitofrontal regions or in the vicinity of large blood vessels, pose the most significant challenge for robust QSM (see Inline Supplementary Fig. S1). Focusing on such regions specifically, it is clear from Fig. 4 that MSDI can effectively constrain undesirable streaking and shadowing artefacts, thus leading to reconstructions with more uniform appearance and more reproducible values than nMEDI.

This study has also confirmed that MSDI's regularisation parameter could be robustly optimised on repeat measurements using the L-curve method. In particular, all optimal *λ*s for MSDI in this study (*i.e.* across multi-echo and single-echo acquisitions at 3T and 7T) were between *λ* = 10^2.4^ (for 1 × 1 × 2 mm^3^, 3T single-GRE) and *λ* = 10^2.7^ (for all high-resolution scans at 7T) – a relatively narrow range, which to some extent was aided by the *TE*·*B*_*0*_ = 60 ms T normalisation step prior to inversion (introduced in this study to harmonise the great dispersion of effective Δ*TE*s moving from broadband multi-echo to long-TE single-GRE acquisitions). It is important to note that curvature estimates from the L-curve method are notoriously volatile, *i.e.* they depend strongly on parameter-sweeping rate and solver-stopping criteria. In this study, we converged on a recipe that was stable across acquisitions, although we cannot rule out unexpected instabilities in future studies. For untested data types or other MSDI settings, the regularisation parameter should again be optimised as the mean (in the log scale, or the median) “L-corner” from several measurements, preferably on multiple subjects.

To date, several spoiled-GRE acquisitions have been proposed for QSM. Therefore, in this study, we assessed the stability of MSDI for a representative range of widely used scan types. We specifically measured scan-rescan QSM variability for a 3T multi-echo GRE sequence, which, except for flip angle, was identical to that used in a previously validated multi-parameter mapping protocol (Callaghan et al., 2015). We contrasted this to the reproducibility of a lower resolution—but fully flow-compensated—3T single-echo scan, which was originally proposed for SWI (Mittal et al., 2009) but has also been used in previous QSM cohort studies (Acosta-Cabronero et al., 2013, 2016, 2017). MSDI returned high quality susceptibility maps for both acquisitions, which were qualitatively similar (see Fig. 5) suggesting that the impact of transmit-related and direct-current offsets on single-echo QSM reproducibility is somewhat small, at least with the present methodology. Interestingly, the reproducibility assessment (contrasting the variability of both scan types) also suggested that QSM from the single-echo acquisition (average σ across the whole brain for five scan repeats on five consecutive days, <σ> = 0.0046, see Fig. 5) might be overall more reproducible than from “3T Multi-Echo” data (<σ> = 0.0074). Elucidating the root cause for this behaviour is beyond the scope of this study, but may be attributable to one or more of the key differences between the two acquisitions, namely (i) single (low bandwidth) echo centred at 20 ms versus eight (broadband) echoes starting at 2.34 ms (the latter thus putatively more sensitive to myelin's short-T_2_* component), (ii) full flow-compensation versus no gradient-moment nulling (*i.e.* multi-echo QSM in this study possibly more vulnerable to flow effects), (iii) two-fold accelerated, 1 × 1 × 2 mm^3^ versus four-fold accelerated, 0.8-mm isotropic voxel resolution (leading to differential scan time, signal-to-noise properties, spatial specificity and bandpass interpolation effects between acquisition types), (iv) true-axial versus 30° field-of-view angulation (*i.e.* multi-echo QSM in this study requiring dipole kernel rotation making it potentially more vulnerable to discretisation errors), and (v) separate-channel GRAPPA reconstruction followed by phase-filtering based coil-combination for single-GRE data versus SENSE-based reconstruction for multi-echo data. The latter might be relevant in that, besides known GRAPPA-SENSE efficiency differences, the coil-combination routine for single-echo data included a discrete Laplacian phase unwrapping step that could have altered the performance of the background-field removal algorithm, and may potentially have resulted in a non-negligible downstream effect on the invertibility of some dipole fields.

Although the greater variance observed for multi-echo QSM clearly warrants a further systematic investigation, additional clues can be inferred from the present dataset. For example, multi-echo QSM was more sensitive (than single, long echo-time QSM) to field offsets originating from the mid-sagittal corpus callosum. Assuming such an effect is putatively driven by myelin's diamagnetism, we could explain both the overall increase in QSM variability and the susceptibility attenuation observed for multi-echo QSM in the corpus striatum (most notably the globus pallidus), in turn traversed by large numbers of myelinated nigrostriatal axons (Morris et al., 1992), as direct consequences of the influence of a short-lived myelin component on short echo-time phase information. This is in agreement with two recent investigations of echo-time dependence that also found absolute susceptibility reductions in the corpus callosum, *i.e.* highly negative susceptibilities for short TE were rapidly attenuated with increasing echo time (Cronin et al., 2017; Sood et al., 2017). Sood et al., however, also predicted QSM attenuation in the globus pallidus for long echo times, which is in conflict with Cronin et al. and these results (Fig. 5). Taken together, these results suggest that the potential QSM dependence on TE is incompletely understood. Meanwhile, therefore, care must be taken when combining GRE data with different effective echo times for group studies.

The high-resolution QSM experiment at high field was also consistent with 3T results in that 7T MSDI reconstructions were overall concordant across scan types (see Fig. 7). To recap, we probed the following aspects: (i) under-sampled versus full k-space data with approximately matched image resolution (∼0.34 × 0.34 × 1.25 mm^3^) – the latter prospectively motion corrected, and (ii) isotropic versus anisotropic image resolutions both using PMC technology and matched for voxel volume (∼0.13 mm^3^) as well as scan time (∼17:30 min). The experiment aimed at: (i) probing whether signal-to-noise ratio differences could drive major QSM offsets when imaging at high spatial resolution, and (ii) shedding new light on the idea that GRE acquisitions with anisotropic voxels might be more suitable for accruing coherent phase from the vasculature than scans with isotropic resolution (Deistung et al., 2008). Although we found relatively strong concordance between 3T and 7T QSM variance distributions, both of which indicated phase measurements from inferior prefrontal, temporal and, generally, deep brain regions are the least robust overall, we did not find any substantive evidence supporting “(i)” in that the observed variability was relatively small, *i.e.* no major systematic offset was observed. However, the present results were in agreement with “(ii)” in that—although differences were small—0.5-mm isotropic resolution QSM returned the greatest overall deviation from the global mean. This is the first such indication with QSM, and also warrants further investigation with more power.

Ultra-high field, high-resolution data yielded MSDI maps with superb anatomical detail. This was driven by several factors including substantial phase-sensitivity and signal-to-noise ratio gains at 7T, and QSM's composite nature – a highly desirable feature in that it makes the technique sensitive to a wide range of tissue properties, hence with potential to detect many neurobiological processes, though conversely it also makes isolating individual susceptibility sources a non-trivial problem. Clearly, new approaches that can decouple different source types from the QSM contrast would be highly desirable. In this study, we did explore the possibility that the spatial filtering properties of Eq. (3) could be used to isolate the vasculature from the present data for VenoMSDI. We explored this conceptually using a combination of morphological and mathematical priors that promoted strong spatial concordance between the distribution of high-pass susceptibilities (HPSM) and MEDI's edge-mask (derived from the signal magnitude). The rationale was that such priors should lead to a source distribution: (i) with highly attenuated susceptibilities where magnitude variations are relatively small (*i.e.* predominantly soft tissue), and (ii) high data-fidelity in regions co-localised with the strongest magnitude gradients (*i.e.* the vasculature), with the regulariser further enforcing 3D continuity for those two compartments. Isolating venous-blood contributions is highly relevant – it would not only enable greater specificity in QSM analyses (by exclusion of vascular contributions), but it could also enable inferences to be made from directly-visualised veins, *e.g.* through oxygen extraction fraction (OEF) measurements. We qualitatively confirmed this hypothesis (see Fig. 7E) and illustrated a direct application to Macro-Vessel Suppressed Susceptibility Mapping (MVSSM, see Fig. 7, Fig. 9 for extended view, and Supplementary Fig. S9 for efficiency-test results), which visually improved QSM's tissue specificity and, potentially, statistical conditioning for whole-brain analyses. Reassuringly, the midline vasculature, for example, was selectively removed whilst the contiguous nonheme—but iron-rich—habenula (small sub-nucleus along the thalamus dorsal surface) was preserved. Further work is warranted to investigate the full potential of VenoMSDI at ultra-high field.

Further focusing on the diencephalon, with the thalamus specifically being a highly relevant use-case for QSM (Deistung et al., 2013), it was also observed that—in addition to habenula—iron-rich pulvinar and an adjacent sub-nucleus from the lateral group (consistent with the location of the ventrocaudal nucleus) contrast highly (paramagnetically) with the surrounding tissue (see Fig. 7G). Notably, these are not the only cases where QSM can help resolve structures where conventional MRI contrasts fall short – for example, anterior, mediodorsal and lateral thalamic subregions, arteries perforating the striatum, the nigral nigrosome-1, and cortical/subcortical iron content variations across the brain are also readily identifiable from high-resolution QSM – all with their expected susceptibility polarity. For a detailed view of high-fidelity HPSM structural capabilities see Fig. 8.

It is worth noting that the QSM contrast is closely related to SWI – a successful MRI technique in current use for a wide range of clinical applications including micro-bleed detection, identification of abnormally calcified tissue, tumour characterisation, and more (Haacke et al., 2009). In the context of the present multi-scale approach, and given that QSM has already been proposed for magnitude-based susceptibility weighting (Gho et al., 2014), we explored specifically whether HPSM could integrate with SWI to further enhance venous contrast by incorporating high-pass susceptibility mapping as an alternative to homodyne-filtered phase distributions. In conventional SWI, a relatively long echo-time (typically 20 ms at 3T) GRE magnitude image is weighted by a linearised, high-pass filtered phase map that is somewhat nonlocal. The potential advantage of using a linearised version of HPSM instead is that it could weight the magnitude image with greater specificity by true-local tissue susceptibilities, which we hypothesised would result in increased SWI sharpness (see Fig. 7J–K and Fig. 10). In SWI, one freely selectable parameter is the kernel window size applied in the Fourier domain to tune the bandpass level of phase filtering used for susceptibility weighting. In MSDI-based SWI, we suggest using short-range HPSM (*i.e. r*_max_ = 2 mm), which can be modulated by an equivalent parameter (Eq. (3)'s *λ* in this case) to enable varying levels of susceptibility weighting to also adapt to different applications. In addition, analogous to minimum-intensity projections from conventional (*i.e.* magnitude-based) SWI (Fig. 10), we showed that maximum-intensity projection maps from optimally regularised HPSM could further enhance the visualisation of small-scale features in contrast to the continuous (healthy) vasculature (Fig. 7, Fig. 11 for extended view).

Finally, a few technical aspects warrant discussion. First, although in this study we demonstrated that MSDI signifies an improvement in terms of accuracy and robustness with respect to nMEDI (the algorithm MSDI is based on), it is also notably slower. This is because the sub-problem defined in Eq. (3) must be solved for several scales with increasing kernel radii, whereas nMEDI solves a similar problem (to that in Eq. (3)) only once. The proposed formulation, however, is not bound to any specific method. It is conceivable that other solvers could result in faster convergence rates or could further improve performance (Milovic et al., 2018). In fact, MSDI's constrained multi-scale problem could be reformulated for efficiency as a single step *e.g.* extending a previously proposed framework (Chatnuntawech et al., 2017).

An interesting difference, however, between Chatnuntawech et al.’s single-step settings and the proposed MSDI implementation is the SMV-kernel radius sampling strategy utilised. Optimised with the Challenge dataset and tested on the remaining *in vivo* experiments (by comparing with nMEDI), this study found that a power-of-two increase in radius up to 16 mm (4 scales) was preferred. Other sampling patterns that were considered were: (i) constant 2-mm steps (8 scales up to 16-mm radius), and (ii) power-of-two sampling up to 8 mm (3 scales) and (iii) up to 32 mm (5 scales). This is an empirical optimisation with relatively low power and no a priori hypothesis that, thus, requires future confirmation.

An additionally important consideration for future work is the assumption of zero QSM mean across the imaging volume, i.e. unreferenced QSM. This is a reasonable assumption in reproducibility experiments and reconstructions challenges where different views of the same object can be expected to have a constant global mean. In addition, past work also found that this global DC uncertainty is only a small adjustment with respect to local aging-related QSM changes (Acosta-Cabronero et al., 2016; Betts et al., 2016). However, there might be scenarios, e.g. in the presence of large haemorrhages or acute iron deposition, where this assumption may break down leading to non-negligible inconsistencies. Recent investigations studied the stability of several referencing methods (Feng et al., 2017; Straub et al., 2017), though in practice, these may not be generalisable *i.e.* reference tissues that are spared in one disease might be unsuitable in other contexts. Referencing to cerebrospinal fluid (CSF) might be a potential solution to this. In fact, a new regulariser for MEDI has been proposed to promote solutions that minimise QSM values in CSF (Liu et al., 2018). This method, however, requires reliable CSF segmentation and exclusion of spurious contributions from the choroid plexus. This extension is called MEDI+0, which is fully compatible with MSDI, though further work is needed to ascertain whether disease-related changes in CSF composition do not confound this strategy.

From a technical standpoint, it is also worth noting the pipeline consisting of BET2 (fractional threshold, 0.1), LBV (approximately two-voxel erosion) and vSMV (one voxel erosion) resulted in highly concordant brain-ROI estimates for all data types in this study, providing accurate and (relatively) artefact-free QSM of the cortex with little tissue loss (see e.g. Suppl. Fig. S3 or Fig. 8). It should be emphasised this is crucial to ensure robust QSM, thus future studies should ensure this, or any other pipeline, are similarly effective with their data type.

A final aspect worth discussing is that this work, as previous nMEDI work did (Liu et al., 2013), found merit in dynamically down-weighting consistency costs for error/artefact propagation control. MSDI extends this concept by selectively rejecting an empirically optimised scale-dependent rule based on the phase second-difference distribution. The present results confirmed that such an adaptive rule is both desirable and effective for a wide range of data types but, notably, other alternative (untested) options exist that need further exploration from a theoretical standpoint.

## 5 Conclusions

Although the future of QSM as a clinical tool is still somewhat uncertain, algorithm calibration studies such as this might help promote its role as a reliable MRI contrast mechanism. In this study, specifically, we presented a new multi-scale approach (MSDI), for which we demonstrated high performance and reproducibility, and illustrated (through examples of HPSM, MVSSM and HPSM-SWI) how selective use of multi-scale information might result in more specific susceptibility-based contrasts, which, in turn, might lead to greater QSM utility, demand and subsequent adoption.

An MSDI implementation is publicly available from the QSMbox software package: https://gitlab.com/acostaj/QSMbox.

## Appendix A Supplementary data

MSDI_paper MSDI_paper
