# Supplementary material for: A robust multi-scale approach to quantitative susceptibility mapping
Source: Neuroimage. 2018 Dec;183:7–24. doi: 10.1016/j.neuroimage.2018.07.065 (PMC6215336; doi:10.1016/j.neuroimage.2018.07.065)
Supplement: MSDI_paper [file mmc1.docx]

**
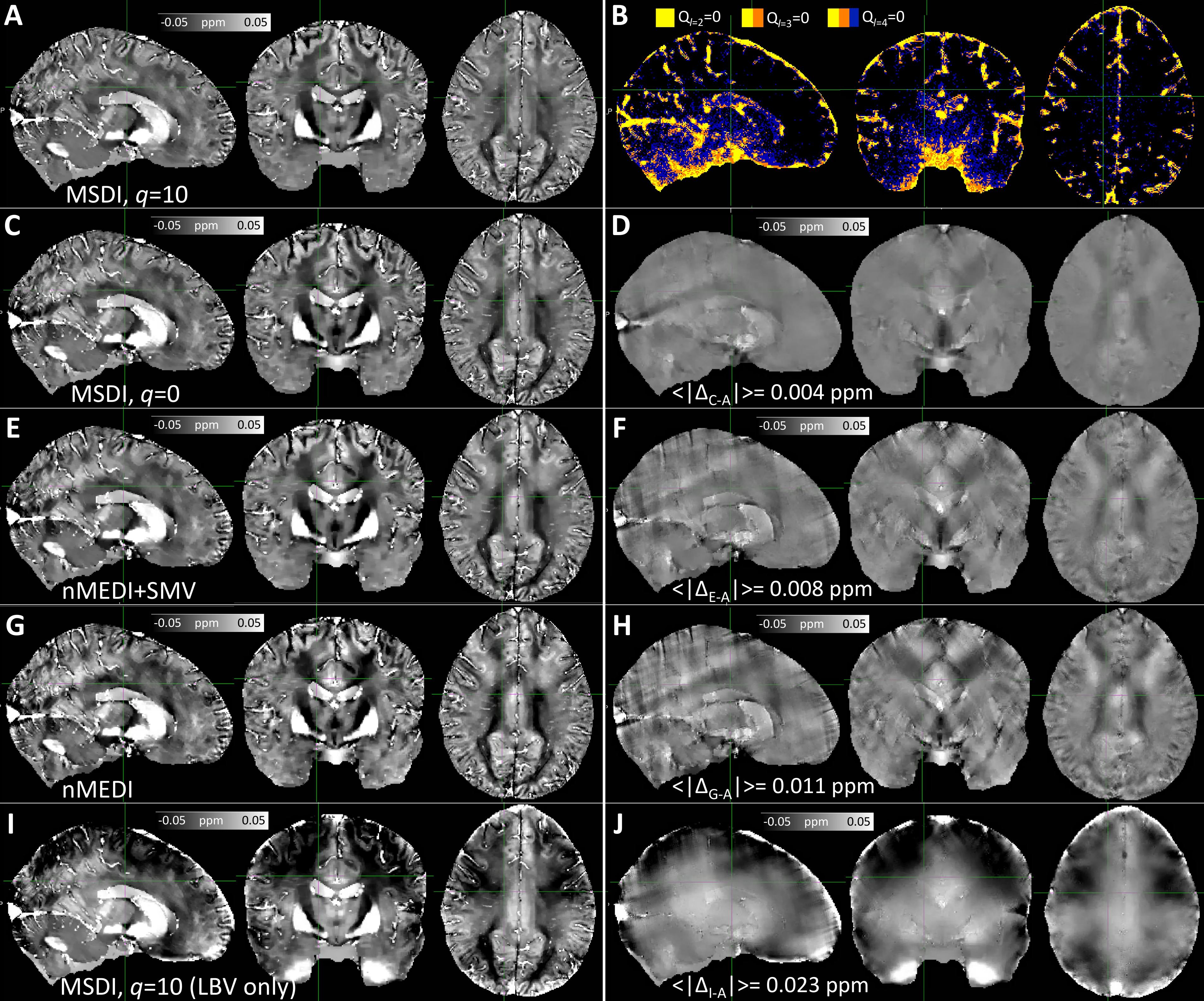
**

**Inline Supplementary Fig. S1**: Schematic illustration of MSDI optimisation. Representative sagittal, coronal and axial slices from a multi-echo 3T dataset for: (A) optimised MSDI with phase pre-processing consisting of Laplacian boundary value (LBV) ([Zhou *et al.*, 2014](#_ENREF_97)) and variable SMV (vSMV) pre-filtering ([Li *et al.*, 2011](#_ENREF_47)); the latter step introduced to correct for LBV’s seemingly incomplete background phase removal (see [I-J] and Inline Supplementary Fig. S2). (B) Scale-dependent extent of consistency masking, *i.e.* *Q_l_* in Eq. 5. (C) MSDI without this masking rule. (D) QSM differential with/without explicit masking of unreliable phases, *i.e.* (C)-(A). (E) nMEDI with single-radius SMV deconvolution. (F) nMEDI_SMV_-optimised MSDI difference map, *i.e.* (E)-(A). (G) nMEDI. (H) nMEDI-optimised MSDI differential, *i.e.* (G)-(A). (I) MSDI without vSMV (optimised parameters otherwise); and (J) difference map illustrating the impact of poor MSDI initialisation , *i.e.* (I)-(A). Summary values reported for difference maps represent global mean absolute differences across the whole brain.


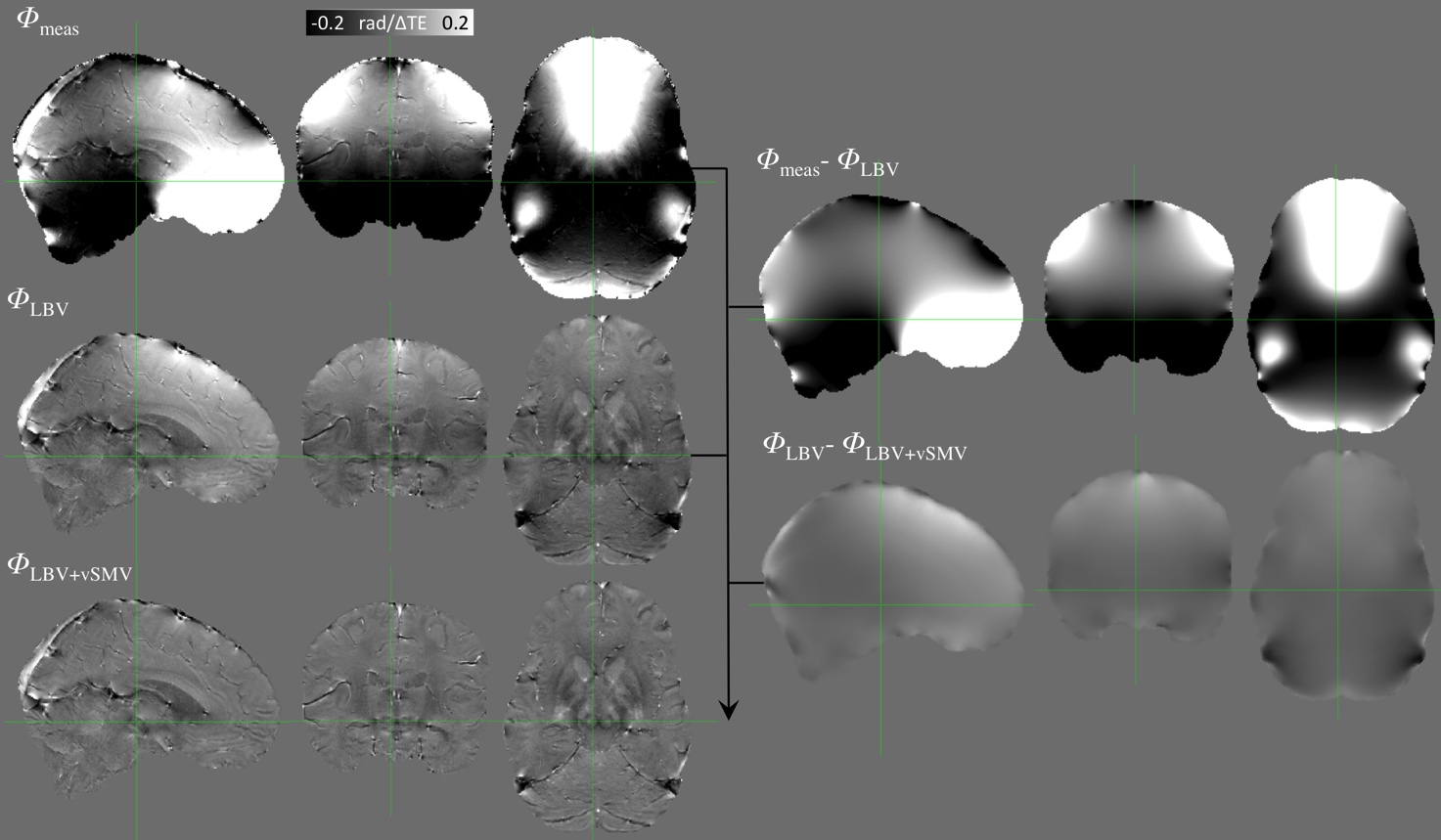


**Inline Supplementary Fig. S2**: Schematic illustration of two-step background-field pre-processing. Unwrapped, echo-combined phase distribution, *Φ*_meas_, filtered in two steps with Laplacian Boundary Value (LBV, available from the MEDI Toolbox, *http://weill.cornell.edu/mri/pages/qsm.html*) and variable SMV algorithms (code adaptation by Job Bouwman, [*https://uk.mathworks.com/matlabcentral/fileexchange/48557-quantitative-susceptibility-mapping-for-mri-part-1*](https://uk.mathworks.com/matlabcentral/fileexchange/48557-quantitative-susceptibility-mapping-for-mri-part-1), of Berkin Bilgic’s original implementation, *http://martinos.org/~berkin/software.html*).


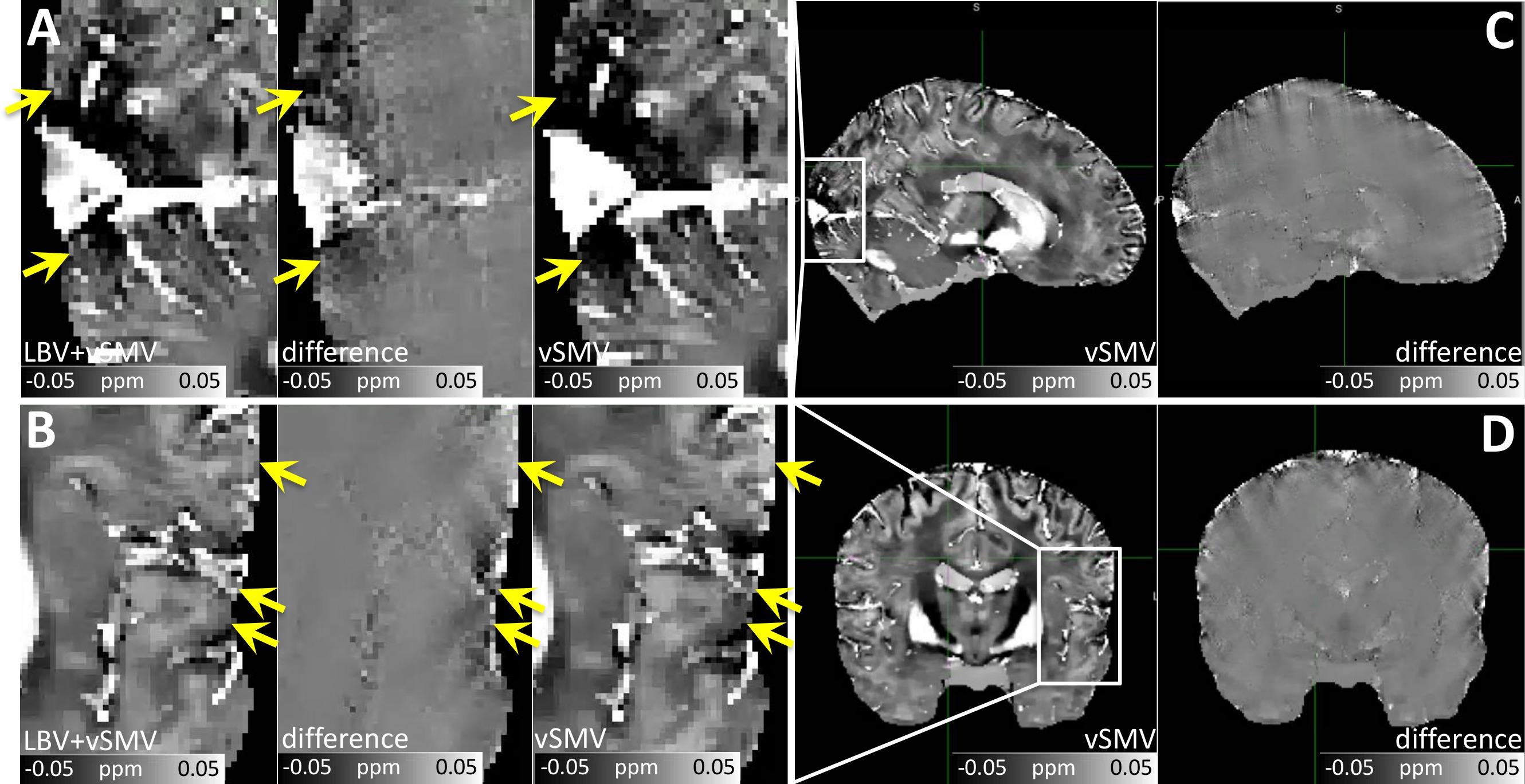


**Inline Supplementary Fig. S3**: Qualitative MSDI improvement with Laplacian Boundary Value (LBV) filtering prior to variable SMV (vSMV). (A) Representative sagittal close-up cuts through occipital and cerebellar regions for: (left) MSDI with two-step pre-processing versus (right) MSDI with vSMV (only), with (middle) representing the QSM difference. (B) The same comparison for a


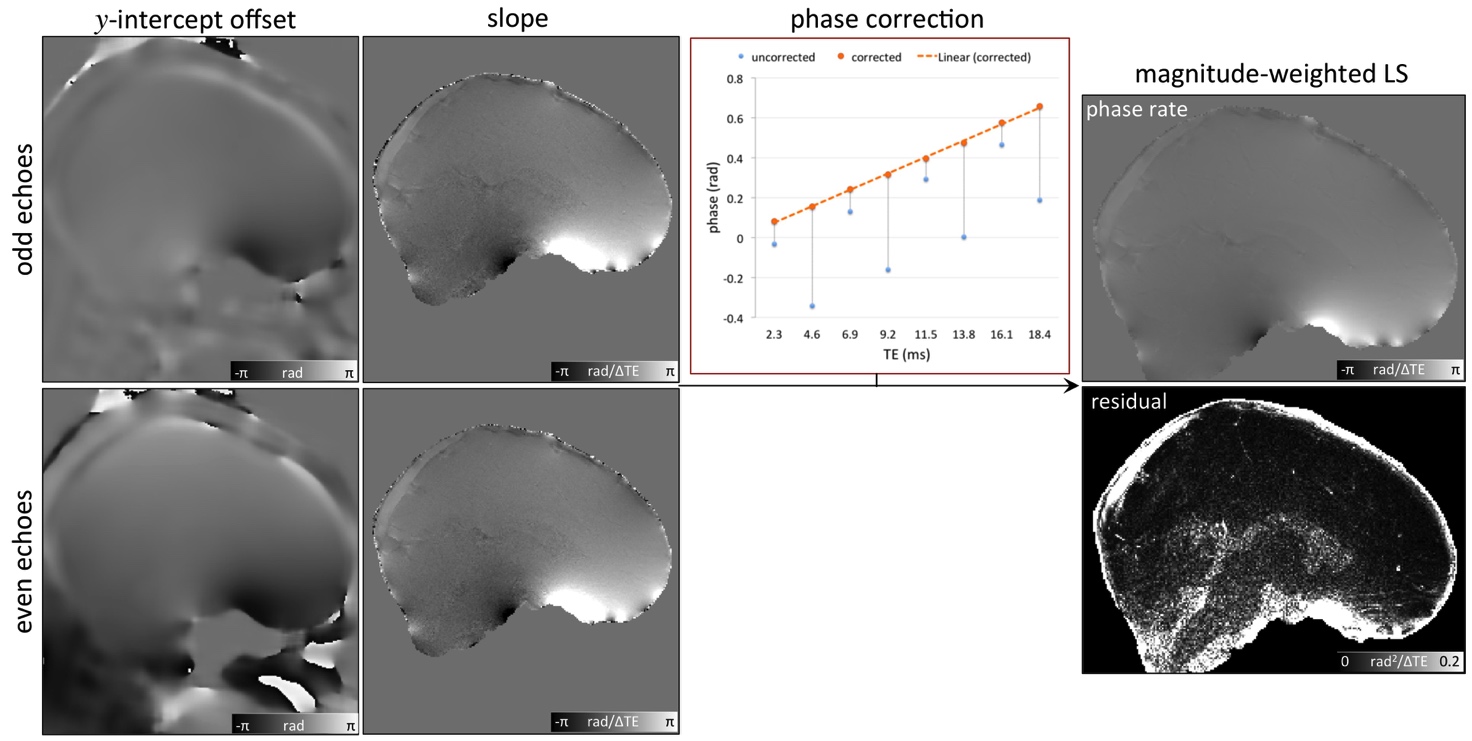


**Inline Supplementary Fig. S4**: Schematic illustration of phase correction and temporal fitting procedures for multi-echo GRE data. These consisted of: (i) *y*-intercept linear extrapolation from the first two odd and even echoes, respectively; (ii) subsequent blurring by convolution with a 16-voxel cubed box filter on the assumption that transmit-related initial phase offsets must vary slowly in 3D space; (iii) initial-offset adjustment to odd-/even-echo phases, respectively; (iv) finally, voxel-wise magnitude-weighted least-squares (LS) regression was performed on the bipolar-readout corrected phase evolution. This routine was based on original code from Dr Hongfu Sun (*https://github.com/sunhongfu/QSM*).


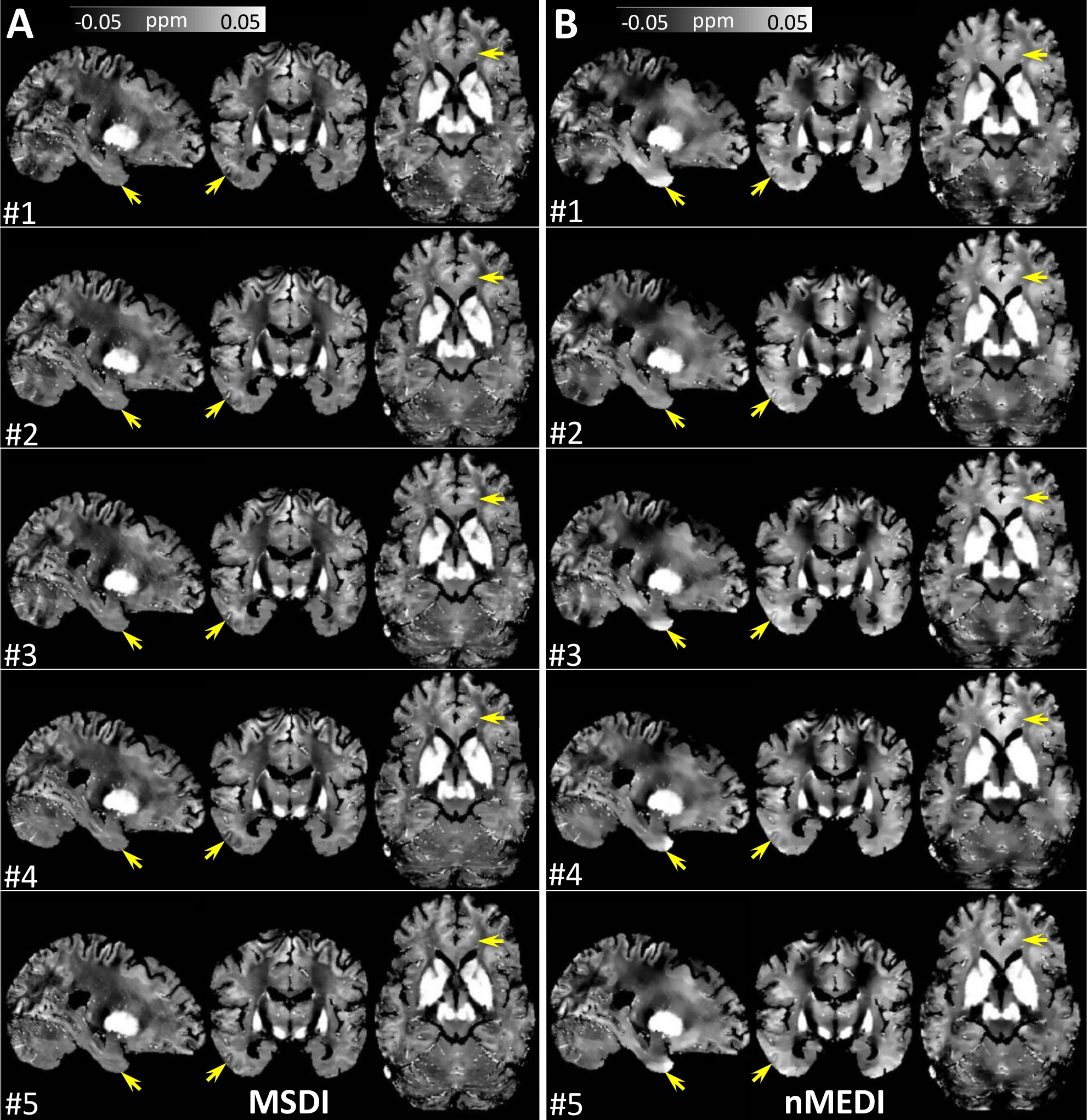


**Inline Supplementary Fig. S5**: (A) MSDI and (B) nMEDI single time-point reconstructions for a reproducibility experiment in which a single subject was scanned on five consecutive days (#1-#5) with a “3T Multi-Echo” GRE sequence. Arrows indicate regions of greater apparent variability (confirmed by greater coefficient of variation in Fig. 4) for nMEDI than for MSDI. Of note, regions of high variance are typically bilateral, though for simplicity arrows are shown unilaterally. All maps are shown in the [-0.05, 0.05] ppm range.


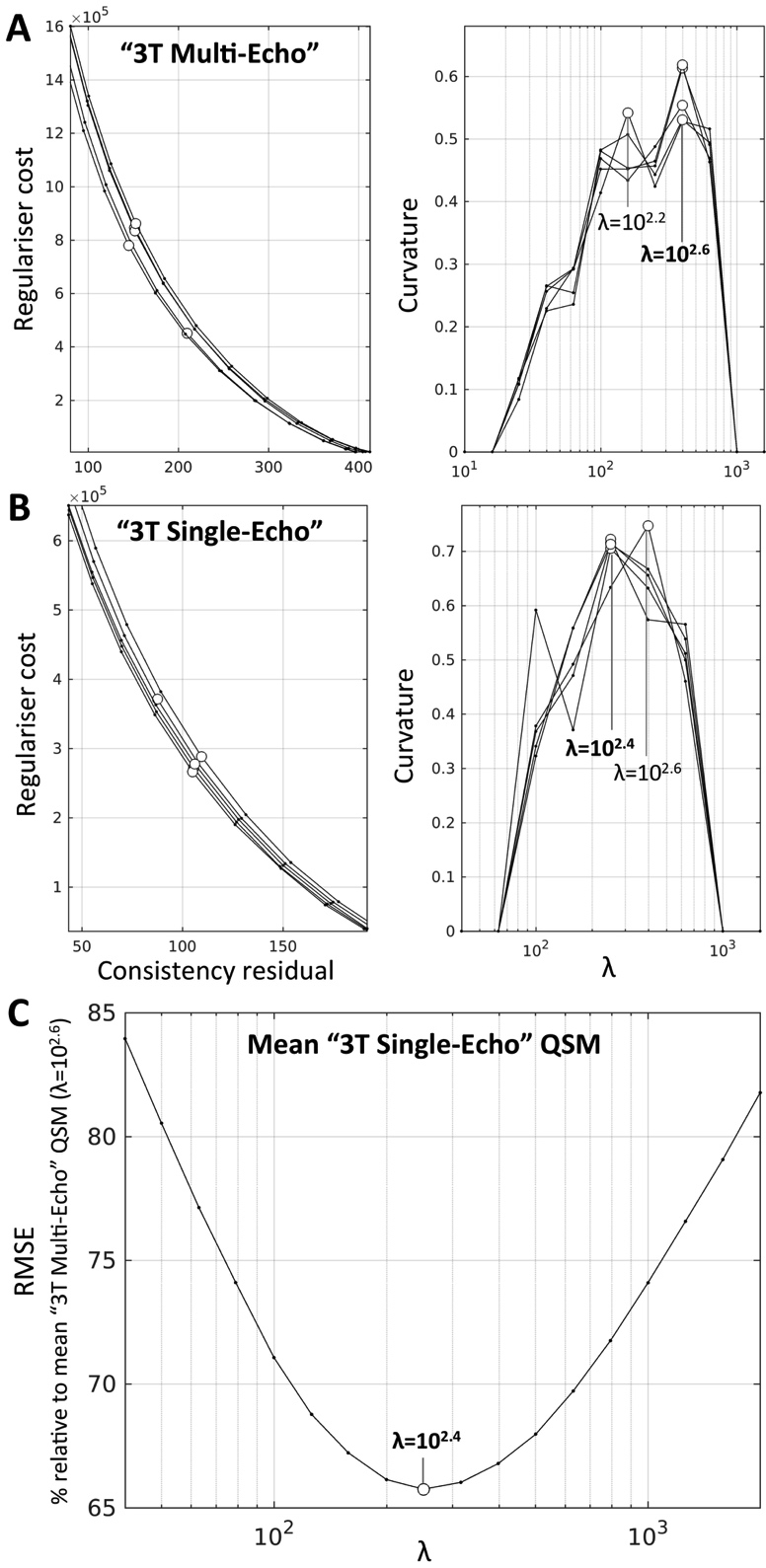


**Inline Supplementary Fig. S6**: L-curve analysis results for five repetitions of (A) “3T Multi-Echo” and (B) “3T Single-Echo” GRE acquisitions on the same subject. (C) Taking QSM averages, confirmation that the (L-curve) optimal regularisation level for single-echo data minimised error differences with respect to the (L-curve optimised) multi-echo MSDI reconstruction.


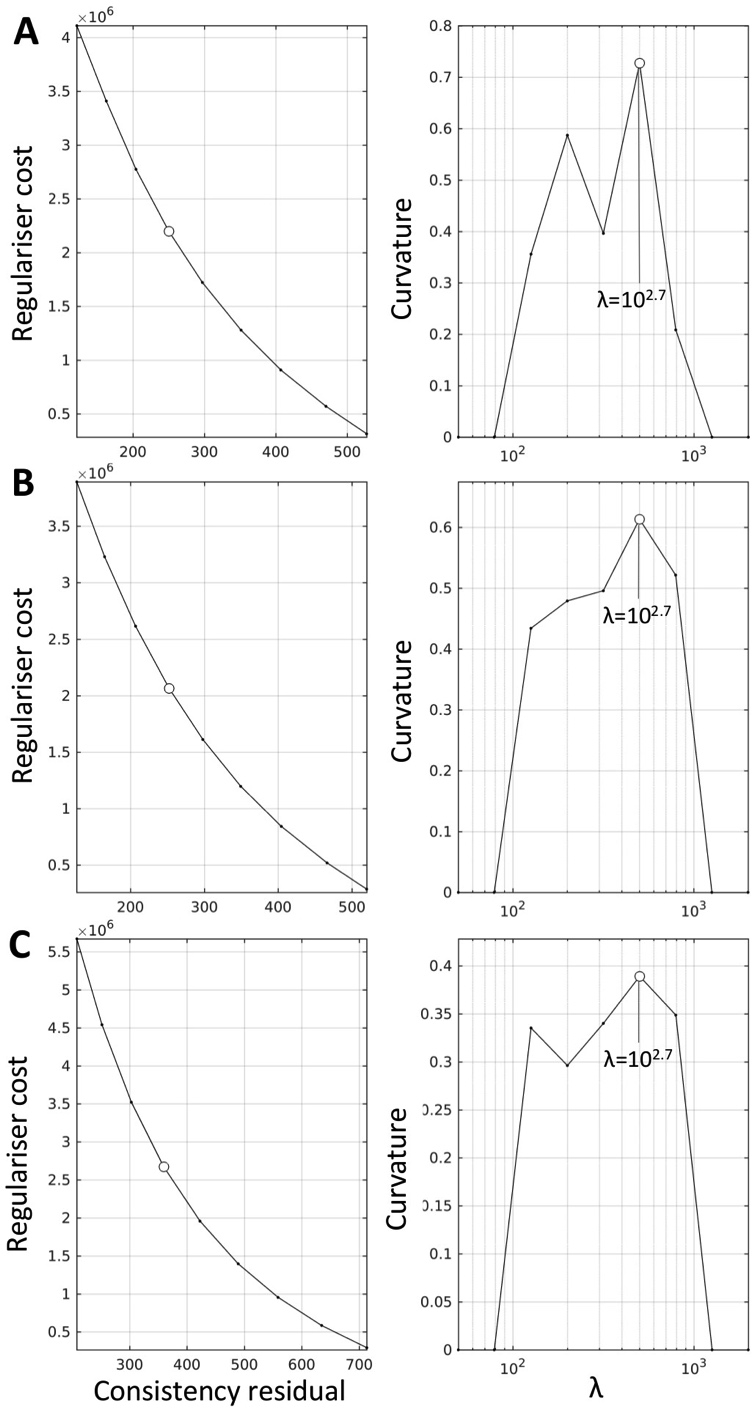


**Inline Supplementary Fig. S7**: L-curve analysis results for (A) “ 7T EUFIND Aniso”, (B) “7T PMC Aniso” and (C) “7T PMC 0.5Iso” data.


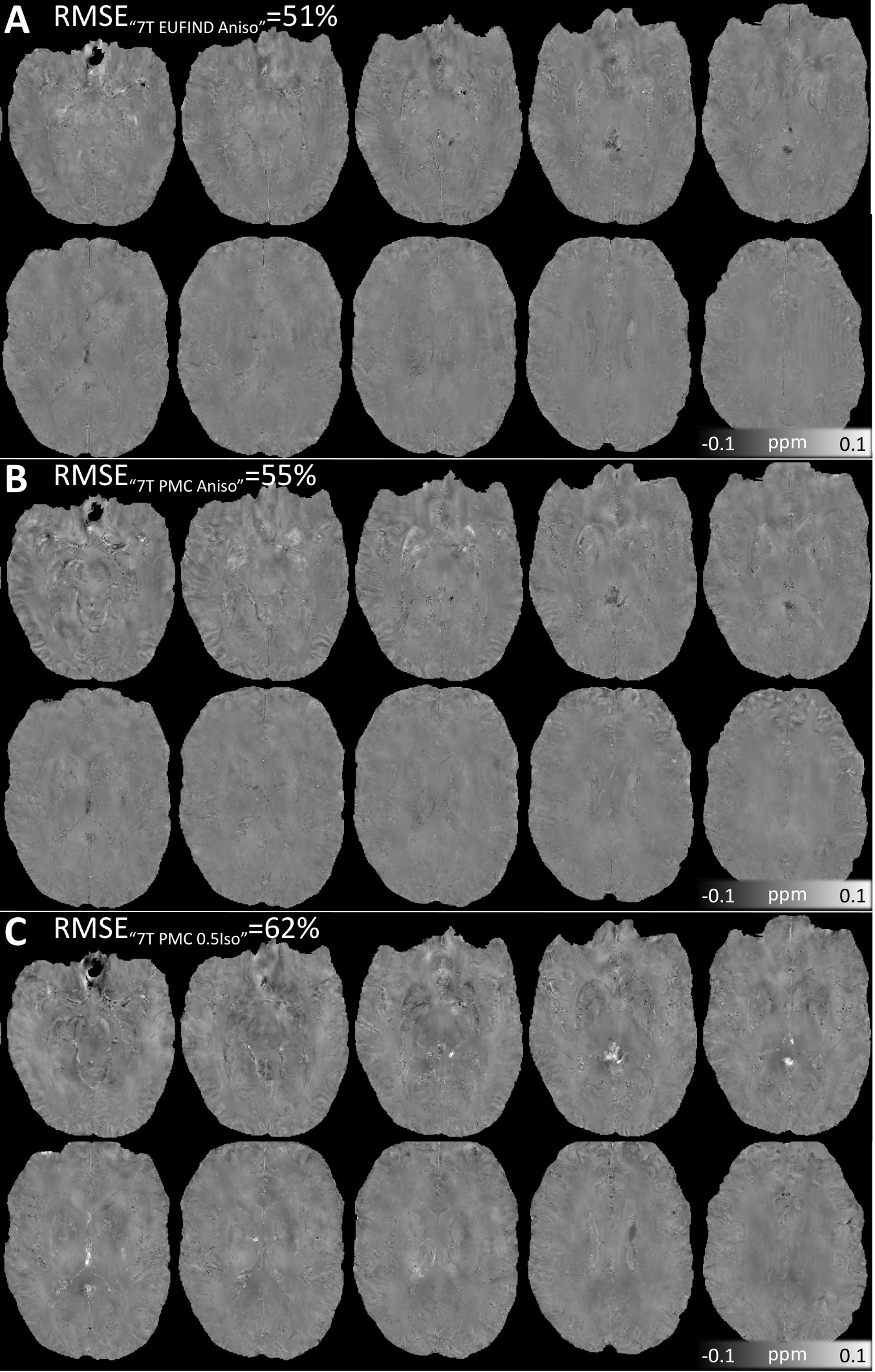


**Inline Supplementary Fig. S8**: MSDI deviations (difference maps and whole-brain root-mean square error calculations from the global mean) for: (A) “ 7T EUFIND Aniso”, (B) “7T PMC Aniso” and (C) “7T PMC 0.5Iso” data.


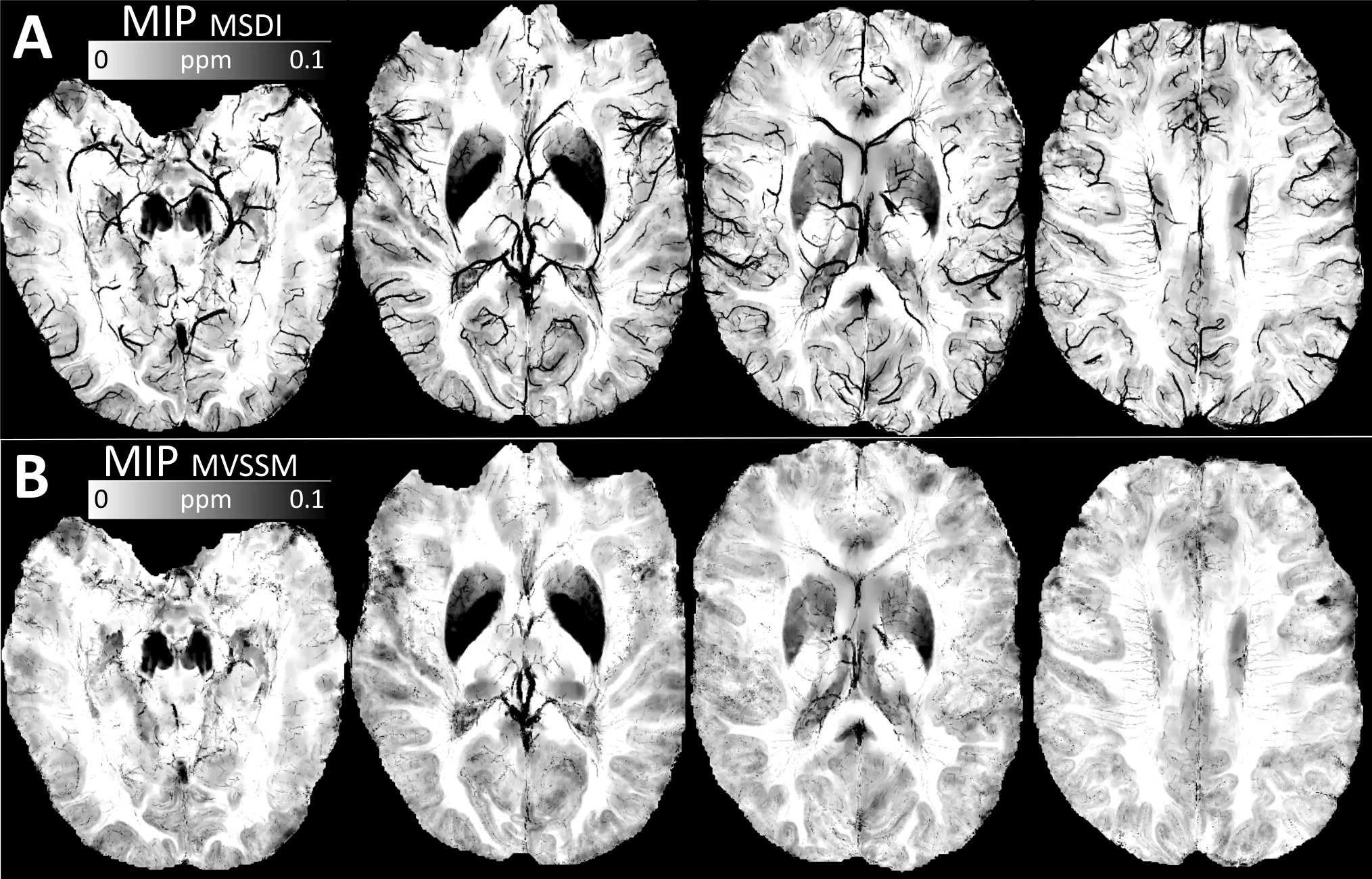


**Inline Supplementary Fig. S9**: Additional illustration of MVSSM’s specificity. The maps shown in (A) for standard MSDI and (B) MVSSM represent maximum-intensity projections (MIP) over 7.5 mm, with reversed colour scale for consistency with conventional mIP_SWI_. Notably, most blood vessels present in (A), particularly those running parallel to the cortical surface, are absent in (B).
